# Supplementary material for: Single-pass STEM-EMCD on a zone axis using a patterned aperture: progress in experimental and data treatment methods
Source: Sci Rep. 2019 Dec 3;9:18170. doi: 10.1038/s41598-019-53373-1 (PMC6890689; doi:10.1038/s41598-019-53373-1)
Supplement: Supplementary file 1 — Supplementary Information [file 41598_2019_53373_MOESM1_ESM.pdf]

Supplementary information for "Single-pass STEM-EMCD on a zone axis using a patterned aperture: progress in experimental and data treatment methods"

Thomas Thersleff[1,*], Linus Schönström[1, 2], Cheuk-Wai Tai[1], Roman Adam[4], Daniel E. Bürgler[4], Claus M. Schneider[4], Shunsuke Muto[3], and Ján Rusz[2]

[1]Stockholm University, Department of Materials and Environmental Chemistry, 10691 Stockholm, Sweden
[2]Uppsala University, Department of Physics and Astronomy, Box 516, 75120 Uppsala, Sweden
[3]Nagoya University, Institute of Materials and Systems for Sustainability, Nagoya 464-8603, Japan
[4]Forschungszentrum Jülich GmbH, Peter Grünberg Institut, D-52425 Jülich, Germany
[*]thomas.thersleff@mmk.su.se

ABSTRACT

This document guides the reader through all of the processing steps necessary to reach the conclusions drawn in the paper it supplements. The purpose is to provide the entire processing workflow necessary to generate the figures. The functions used in this script are provided alongside the data and are available on Zenodo (DOI: 10.5281/zenodo.3361582).

Table of Contents

Introduction to this script and the supplementary information . . . 1
Description of the files ▪ Dependencies ▪ To run this script
Import the EELS data . . . 3
Generate the axis vectors in real space and reciprocal space ▪ Load the 4D EELS dataset ▪ Load the tag file with time stamps ▪ Identify good frames ▪ Extract good frames from the raw data and visualize it
Import STEM-DP datacube . . . 4
Explore the STEM-DP datacube ▪ Generate orientation masks ▪ CBED patterns for different orientations
4D EELS pretreatment . . . 16
X-Ray spike identification and removal ▪ Rough energy alignment (correcting for the drift-tube shift) ▪ Correct for outliers ▪ Apply the rough ISOmap and visualize it ▪ Fine energy drift correction ▪ Apply the complete energy drift offset ▪ Interpolation to 4D datacube
Candidate EMCD signal extraction: . . . 25
Necessary function handles ▪ Introduction to the `optimMLMS.m` function ▪ Using `mask.orient01` (close to zone axis) and restricted q_y ▪ Using `mask.orient02` (tilted away from zone axis) and restricted q_y ▪ Using `mask.grain` and all q_y values ▪ Using `mask.grain` (integral over all orientations) and restricted q_y ▪ EMCD q-maps

Introduction to this script and the supplementary information

This document guides the reader through all of the processing steps necessary to reach the conclusions drawn in the paper it supplements. It was written using the Matlab Live Script utility in version R2018b. The purpose is to introduce the code used in the paper and reproduce the entire processing workflow necessary to generate the figures. The functions used in this script are provided alongside the data and are available through the Zenodo website at the following URL or using the digital object identifier listed below:

https://zenodo.org/record/3361582

10.5281/zenodo.3361582

Description of the files

There are four data files that accompany this script. Two of the files are the raw data as captured in the TEM and two of them are timestamp metadata. The two raw data files are saved in Gatan's proprietary `.dm4` format, while the two timestamp files (which allow for synchronizing the data frame to the probe position) are saved in Gatan's proprietary `.gtg` persistent tag structure format. The timestamp files are used to arrange the data into a 4D datacube, which subsequently allows for the STEM-DP and 4D-EELS datasets to be correlated through their common spatial dimensions.

- `EELS4D_Movie.dm4`: Image stack of all of the 2D EELS frames that were captured from the Ultrascan camera running in "view" mode using the hook-up script written by Linus Schönström and a modification of the frame recorder written by Vincent Hou (see the manuscript for details).
- `DP_Movie.dm4`: Image stack of all of the CBED frames that were captured from the Orius camera running in "view" mode using the hook-up script written by Linus Schönström and a modification of the frame recorder written by Vincent Hou (see the manuscript for details).
- `STEMDP_TimeStamps.gtg`: Collection of time stamps for the STEM-DP experiment. It goes along with the file `DP_Movie.dm4`.
- `EELS4D_TimeStamps.gtg`: Collection of time stamps for the 4D EELS experiment. It goes along with the file `EELS4D_Movie.dm4`.

Dependencies

The first public release of this script and the accompanying code is v1.0. This script assumes that this release is being used. Later releases may break the code.

The use of this script relies on a number of third party software packages. First and foremost, both a recent version of Matlab as well as the optimization toolbox is required. These are commercial packages that must be purchased from The Mathworks, Inc. This script was written in version R2018b.

The raw data were recorded and are thus provided in a proprietary format developed by Gatan, Inc. Andreas Korinek's dmread.m script, which, at the time of writing, can be downloaded from the Matlab file exchange, is used to read and import this format into Matlab. It is included in the package for this work in unmodified form aside from a small bug fix that is commented.

The divergent colormaps come from a Matlab implentation BrewerMap, which is based off of the colormaps developed by Cynthia Brewer (http://colorbrewer.org). A copy of this library is included in the repository with this code.

For false-color sequential colormap (with the exception of grayscale), the MatPlotLib 2.0 default colormap "inferno," as implemented in Matlab in the Colormaps-from-MatPlotLib2.0 library, is used. An unmodified copy of this library is included in this repository.

The data visualization functions `viewESI.m` and `sliceViewer.m` both make use of version 2.3.3 of the GUI Layout Toolbox by David Sampson of The Mathworks, Inc. It can be either installed as a Matlab addon or downloaded from the Mathworks file exchange.

Sub-channel shifts in `applyISOmap.m` use version 1.1.0.0 of the package "Efficient subpixel image registration by cross-correlation" by Manuel Guizar et. al. This code is based off of the citation below and is provided along with this script.

Manuel Guizar-Sicairos, Samuel T. Thurman, and James R. Fienup, "Efficient subpixel image registration algorithms," Opt. Lett. 33, 156-158 (2008).

To run this script

1. Create a new folder and copy the data (`.dm4` and `.gtg` files) into it.
2. Copy this script into the base folder*.
3. Copy all of the dependencies into their respective subfolders*.
4. Open up Matlab and navigate to this folder within the developer environment
5. Add all of the provided scripts and the dependencies listed above to the Matlab path. If the dependencies are subfolders of the current path, this can be accomplished with the code: `addpath(genpath(pwd));`
6. Run this script, selecting the appropriate `.dm4` files when prompted.

* With git installed, steps 2 and 3 can be accomplished simply by opening up a git bash in the base folder and then cloning the github repository with the following command:

```
git clone https://github.com/tthersleff/Single-pass-EMCD-analysis-code.git -b v1.0
```

Import the EELS data

In this section, the stack of 2D EELS frames is imported into Matlab and assembled into a 3D datacube. This 3D datacube will have dimensions $\Delta E \times q_y \times N_{px}$ where ΔE represents the EELS dispersion dimension, q_y represents the momentum transfer dimension in the transverse axis (non-dispersion axis in EELS), and N_{px} represents the linear index of the spatial dimension. Linear index in this case refers to the sequential frame number of the 2D EELS acquisition. Assembling this dimension into the two separate x and y dimensions will not take place until the subsection Interpolation to 4D Datacube. The information required to achieve this assembly is saved in variable `ts`.

Generate the axis vectors in real space and reciprocal space

```
dq = 12.37/21.4; %mrad.  Sample of reciprocal space
qx = ((0:255) - 128) .* dq; %mrad.  Reciprocal space vector for STEM-DP dataset
x = ((0:79) - 40) .* 6.25; %nm.  Real space vector

% The vector qy will be used for the 2D EELS frames.  There are 32 channels
% and the spacing between them is about 2.2 mrad.  We center it at zero in
% the middle of the vector
dqy = 2.2; %mrad
qy = ((1:32) - (32/2)) .* dqy;
```

Load the 4D EELS dataset

The persistent tag structure of .dm4 file is imported as variable `tg`. The data are extracted and reshaped into a 3D datacube in variable `A`.

```
tg = dmread('EELS4D_Movie.dm4');
A = reshape(tg.ImageList.Unnamed1.ImageData.Data.Value, ...
            tg.ImageList.Unnamed1.ImageData.Dimensions.Unnamed0.Value, ...
            tg.ImageList.Unnamed1.ImageData.Dimensions.Unnamed1.Value, ...
            tg.ImageList.Unnamed1.ImageData.Dimensions.Unnamed2.Value);
A = double(A);
size(A)
```

```
ans = 1x3
        2048        32        8000
```

```
clear tg; % No longer needed, but has a large memory footprint
```

Load the tag file with time stamps

The function `getDiffImage` is used to import only the timestamps. Construction of the 4D EELS datacube occurs only after the EELS pretreatment is complete. Thus, a dummy variable (~) suppresses the first output.

```
% When prompted, choose the file "EELS4D_Movie.dm4"
[~, ts] = getDiffImage('EELS4D_TimeStamps.gtg');
```

```
Found and removed 1 bad pixel timestamp(s)
ESI was paused 0 time(s).  Expecting 1 DP stack(s)
```

Identify good frames

This also removes the frames acquired while the probe was not scanning at the beginning and end, as well as the empty frames that were allocated during the image container generation but not filled since the frame grabber was stopped prematurely.

```
goodframes = ts.framegrab >= ts.pxbegin(2) & ts.framegrab <= ts.pxend(end);
fprintf(1, 'There were %d good frames captured', sum(goodframes));
```

```
There were 5973 good frames captured
```

Extract good frames from the raw data and visualize it

```
A2 = A(:, :, goodframes);
try
   sliceViewer(permute(A2, [3 2 1]));
   colormap inferno(1024);
    axis normal;
catch
    fprintf(1, 'Ensure that the GUI Layout Toolbox is installed');
end
```

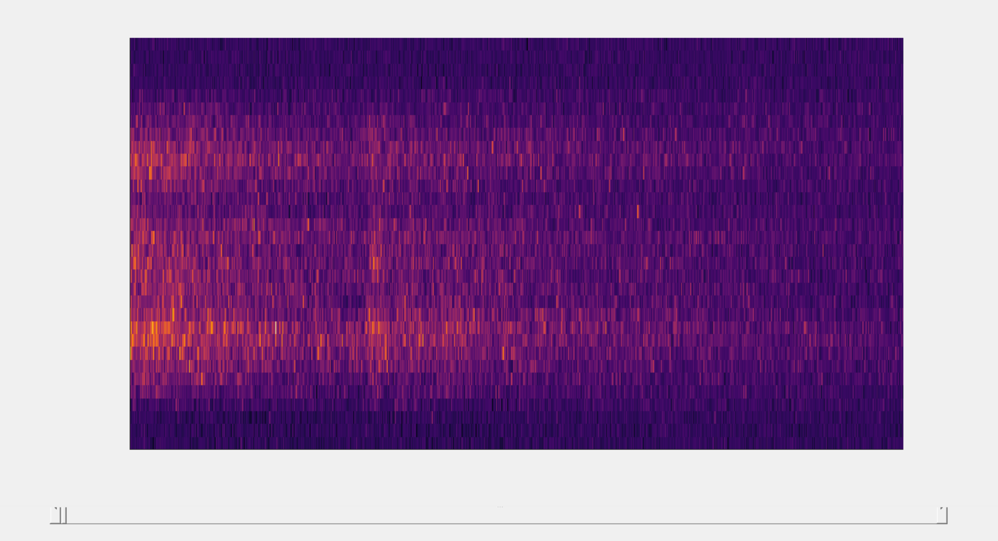

Import STEM-DP datacube

Unlike with the EELS data, the stack of CBED patterns can be directly assemblied into the 4D STEM-DP datacube, as no pre-treatment is necessary.

```
% When prompted, choose the file "DP_Movie.dm4"
[STEMDP, tsDP] = getDiffImage('STEMDP_TimeStamps.gtg');
```

```
Found and removed 0 bad pixel timestamp(s)
ESI was paused 0 time(s).  Expecting 1 DP stack(s)
```

```
% Due to the way that the data are recorded, a permutation is necessary in
% Matlab
STEMDP = permute(STEMDP, [2 1 4 3]);
```

The "map histogram" reveals how many CBED patterns were assigned to each pixel:

```
tsDP.maphistogram = tsDP.maphistogram'; % Permutation is required, as discussed
    above

% Figure generation
figure('Color','w');
imagesc(x, x, tsDP.maphistogram);
axis image;
set(gca, 'colormap', gray(max(tsDP.maphistogram(:))+1));
colorbar;
title('CBED frames per pixel')
set(gca, 'FontSize', 16);
set(findobj(gcf, 'Type', 'colorbar'), 'Ticks', [0:max(tsDP.maphistogram(:))]);
xlabel('Distance [nm]');
ylabel('Distance [nm]');
```

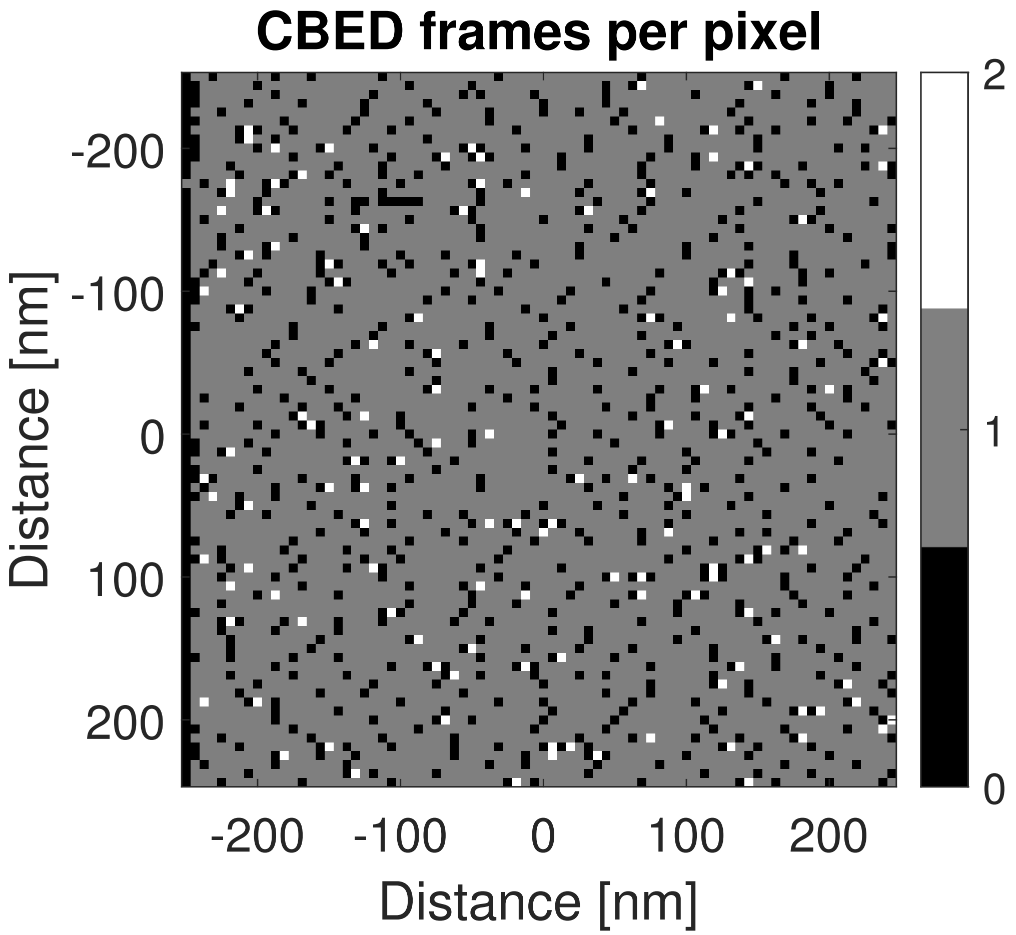

Explore the STEM-DP datacube

The code provided with this script does not include a Matlab function to explore 4D Datasets effectively. We recommend that one exports the datacube to Hyperspy and uses the PyXem package. For now, the datacube can be programatically explored by generating virtual HAADF images, to ensure that the patterns were properly imported. Orientation masks using virtual dark field samples will then also be created. Proper placement of the virtual apertures was empirically determined by interactively exploring the data in PyXem. Finally, a "grain" mask that represents the entire iron grain under investigation will also be produced. These masks reproduce the Region Of Interest (ROI) figure presented in the paper.

```
[rx, ry] = meshgrid(qx, qx);
% HAADF range > 40 mrad
Rc = sqrt((rx.^2 + ry.^2)) > 40 & sqrt((rx.^2 + ry.^2)) < qx(end);

% Plot mean CBED pattern
figure('Color','w');
imagesc(qx, qx, mean(mean(STEMDP, 4), 3));
axis image;
colorbar;
title('Mean CBED pattern')
set(gca, 'FontSize', 16);
xlabel('q_x [mrad]');
ylabel('q_y [mrad]');
% imGamma puts a gamma curve on the grayscale colormap
set(gca, 'colormap', imGamma(gray(10000), 0.3));
```

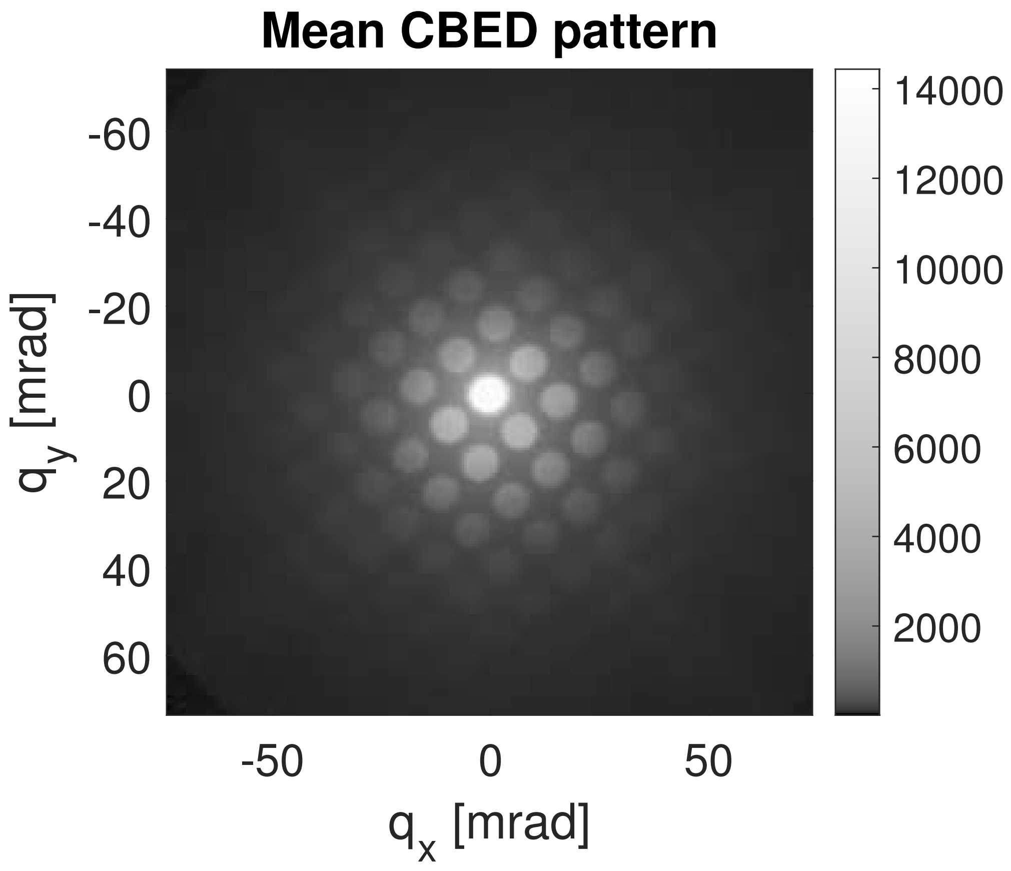


```
% Plot virtual HAADF detector
```

```
figure('Color','w');
imagesc(qx, qx, Rc);
set(gca, 'colormap', gray(2));
axis image;
colorbar;
title('Virtual HAADF detector')
set(gca, 'FontSize', 16);
set(findobj(gcf, 'Type', 'colorbar'), ...
    'Ticks', [0.25 0.75], 'TickLabels', {'Inactive', 'Active'}, ...
    'TickLength', 0);
xlabel('q_x [mrad]');
ylabel('q_y [mrad]');
```

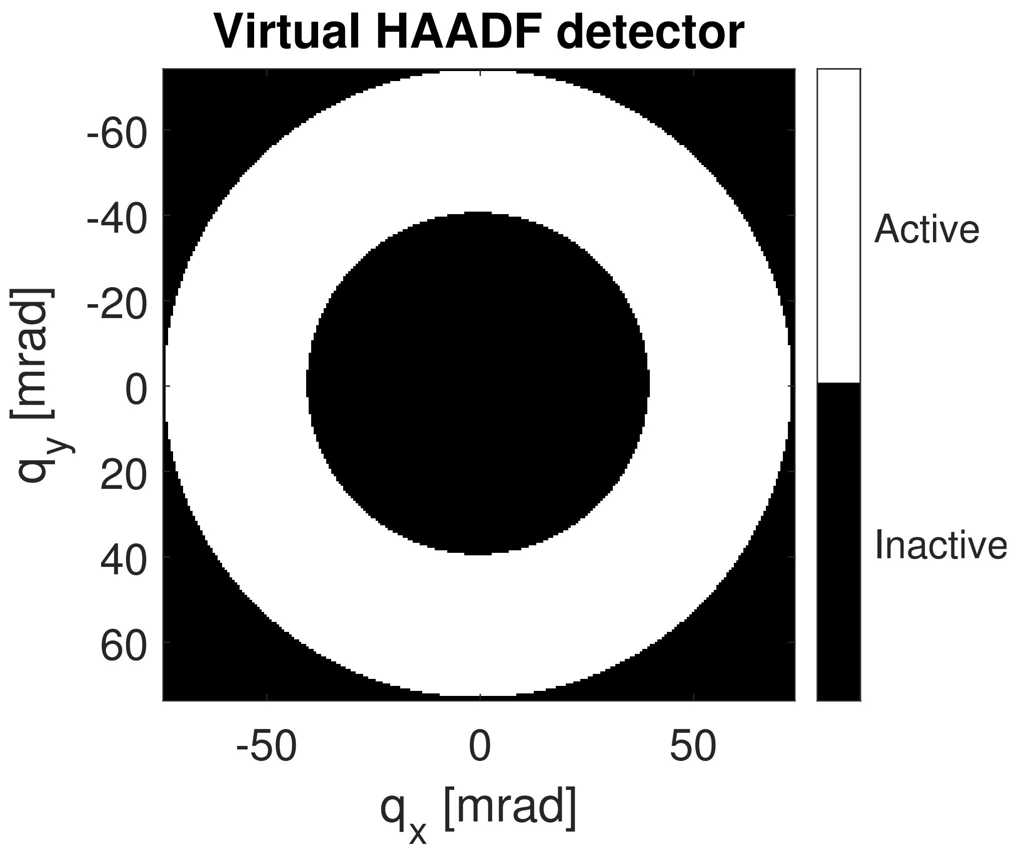


```
% Plot the virtual HAADF image (in real space!)
haadf = squeeze(sum(sum(STEMDP .* Rc, 2), 1));
figure('Color','w');
imagesc(x, x, haadf);
colormap gray;
axis image;
colorbar;
title('Virtual HAADF image')
set(gca, 'FontSize', 16);
xlabel('Distance [nm]');
ylabel('Distance [nm]');
```

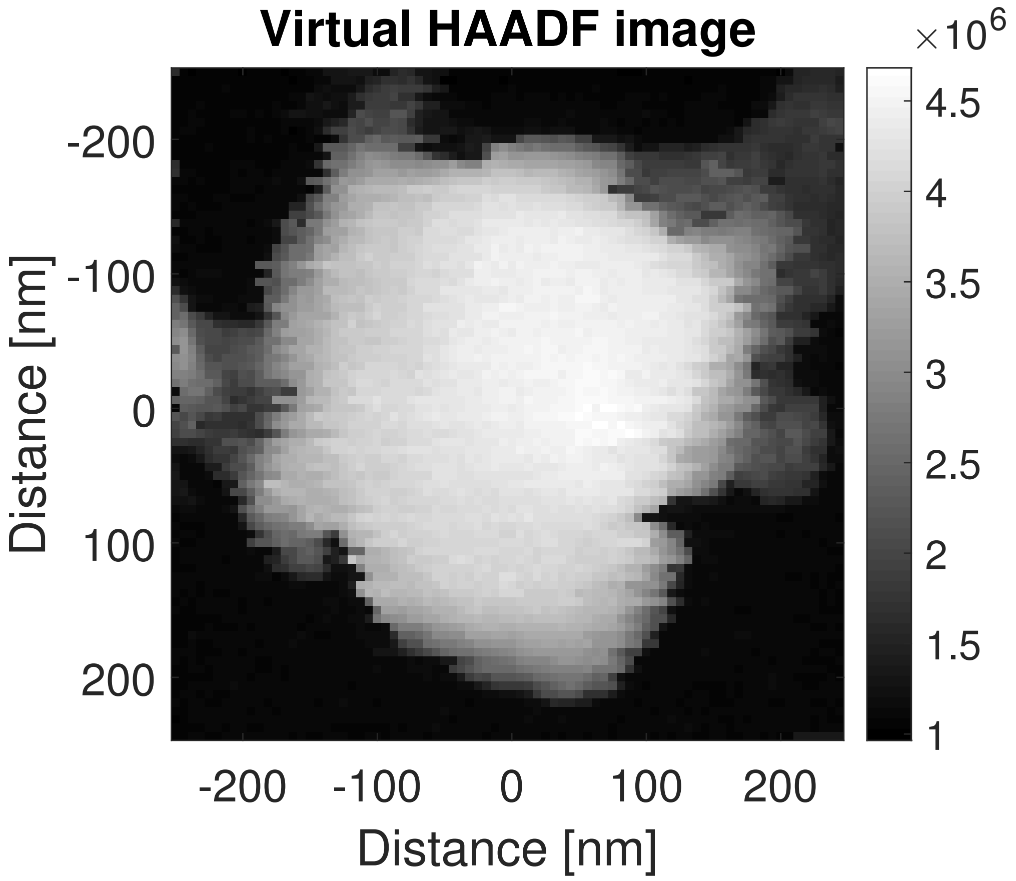

Generate orientation masks

In this section, we use the STEM-DP data to generate orientation masks. If one browses the data (for example using PyXem), one sees that some regions of the iron grain are closer to the [1 0 0] zone axis orientation than others. To estimate this, we look at a Kikuchi line that varies quite a bit as the orientation changes. This will give us an estimate of a zone-axis orientation vs. an orientation that is tilted further away.

```
% Virtual Bright Field
% Aperture placement is a box using indices 125:135 in x and y.  Recall
% that this was empirically determined
vbf = squeeze(sum(sum(STEMDP(125:135, 125:135, :, :), 1), 2));

% Plot the VBF image
figure('Color','w');
imagesc(x, x, vbf);
colormap gray;
axis image;
colorbar;
title('Virtual BF image')
set(gca, 'FontSize', 16);
xlabel('Distance [nm]');
ylabel('Distance [nm]');
```

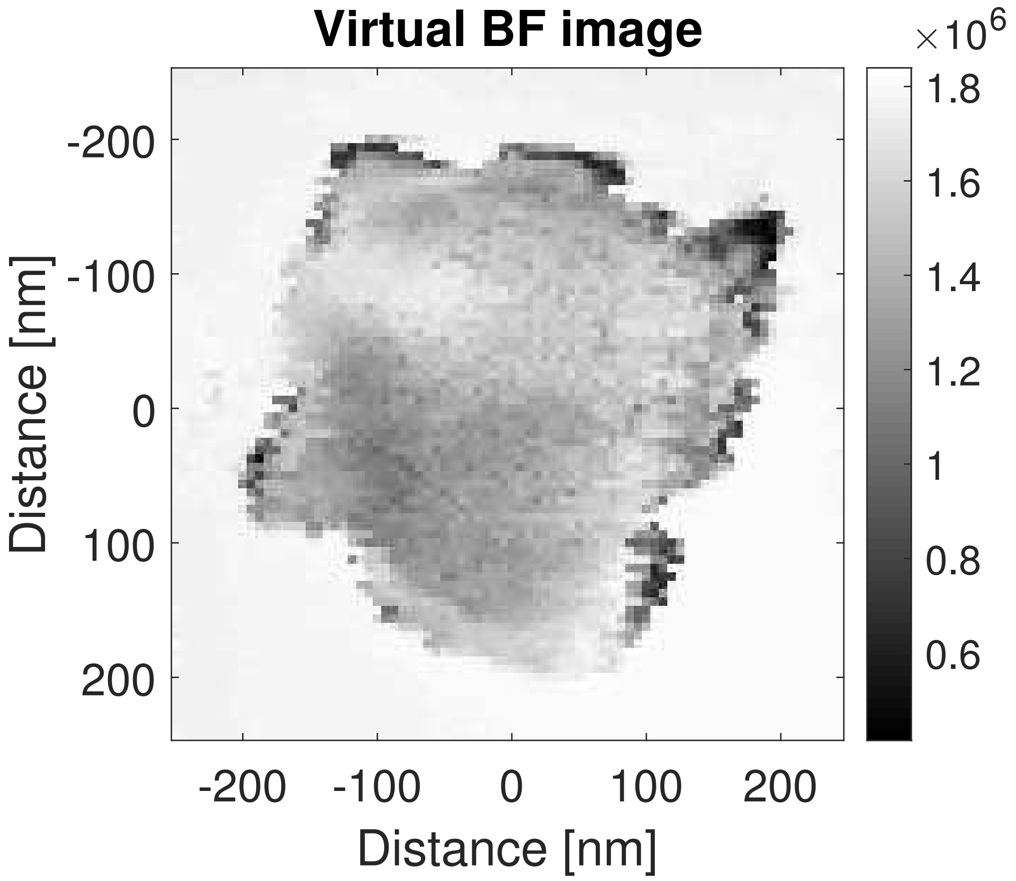


```
% Virtual Dark Field
% Aperture placement is a box from 205:216 and 18:26.  These values were
% determined empirically by exploring the data in PyXem and are intended to
% capture variation of a Kikuchi line set
vdf = squeeze(sum(sum(STEMDP(205:216, 18:26, :, :), 1), 2));

% Plot the VDF image
figure('Color','w');
imagesc(x, x, vdf);
colormap gray;
axis image;
colorbar;
title('Virtual DF image')
set(gca, 'FontSize', 16);
xlabel('Distance [nm]');
ylabel('Distance [nm]');
```

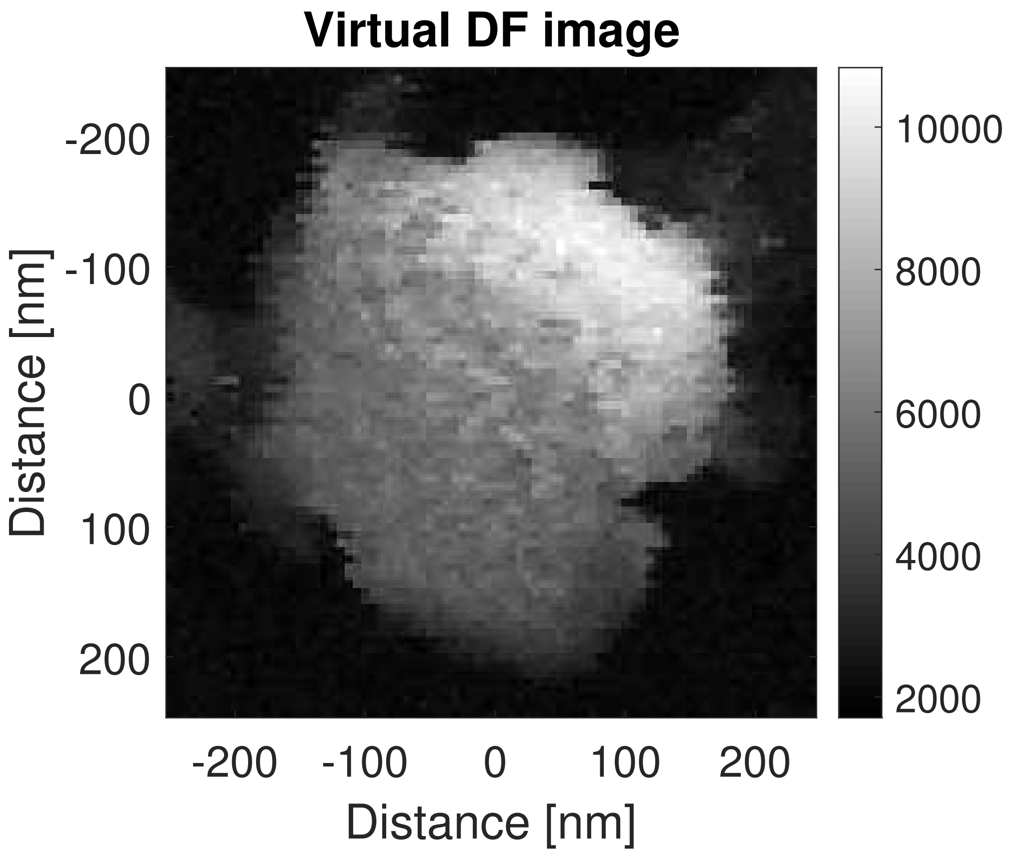


```
% Mask: Full grain
% The thresholding values were emperically determined and are intended to
% isolate the central grain.  The principle is the loss of scattering
% intensity in the central disk due to the high symmetry.  This excludes
% neighboring iron grains that are in lower symmetry orientations.
mask.grain = (vbf < 1.8e6 & vbf > 1e6) & haadf > 3.5e6;

% Plot the grain mask
figure('Color','w');
imagesc(x, x, mask.grain);
set(gca, 'Colormap', gray(2));
colorbar;
set(findobj(gcf, 'Type', 'colorbar'), ...
    'Ticks', [0.25 0.75], 'TickLabels', {'False', 'True'}, ...
    'TickLength', 0);
axis image;
title('Mask: Grain')
set(gca, 'FontSize', 16);
xlabel('Distance [nm]');
ylabel('Distance [nm]');
```

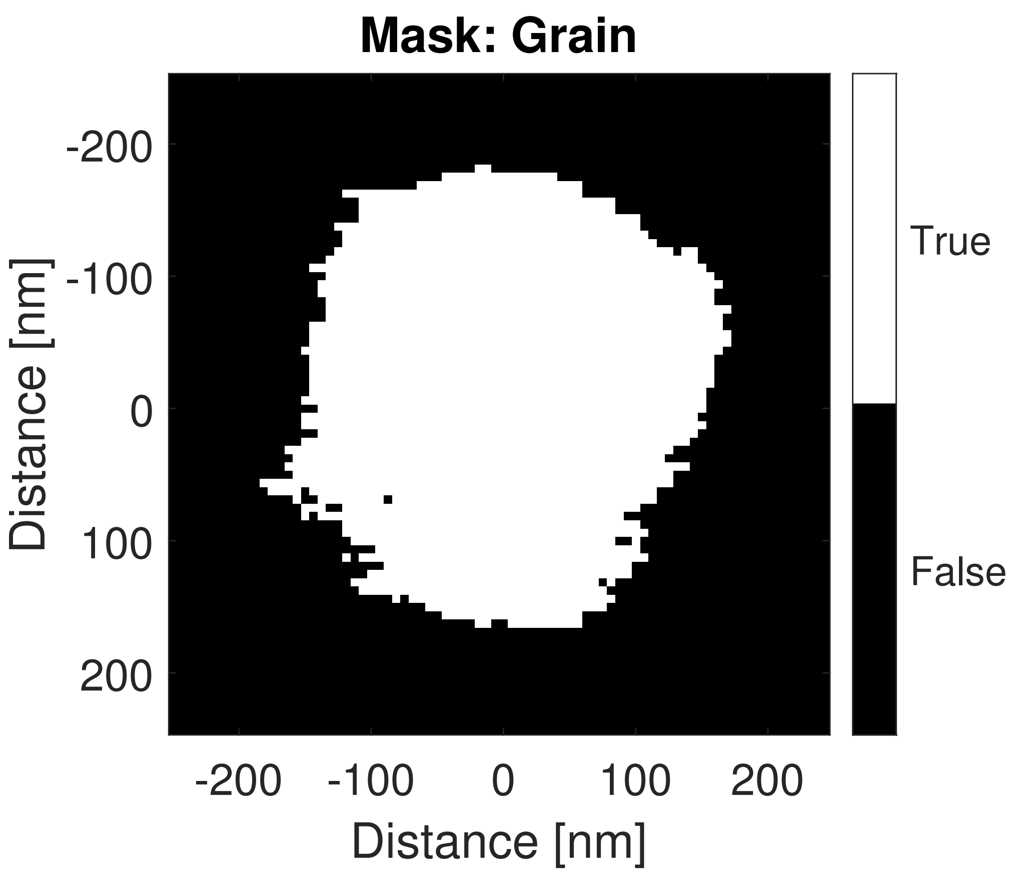


```
% Mask: Orient 01
% The thresholding values for these orientation masks were likewise
% determined empirically.  They are furthermore constrained to the "grain"
% region using the logical AND operator
mask.orient1 = vdf > 6000 & mask.grain;

% Plot the mask for orientation 1 (closer to the zone axis)
figure('Color','w');
imagesc(x, x, mask.orient1);
set(gca, 'Colormap', gray(2));
colorbar;
set(findobj(gcf, 'Type', 'colorbar'), ...
    'Ticks', [0.25 0.75], 'TickLabels', {'False', 'True'}, ...
    'TickLength', 0);
axis image;
title('Mask: Orient 01')
set(gca, 'FontSize', 16);
xlabel('Distance [nm]');
ylabel('Distance [nm]');
```

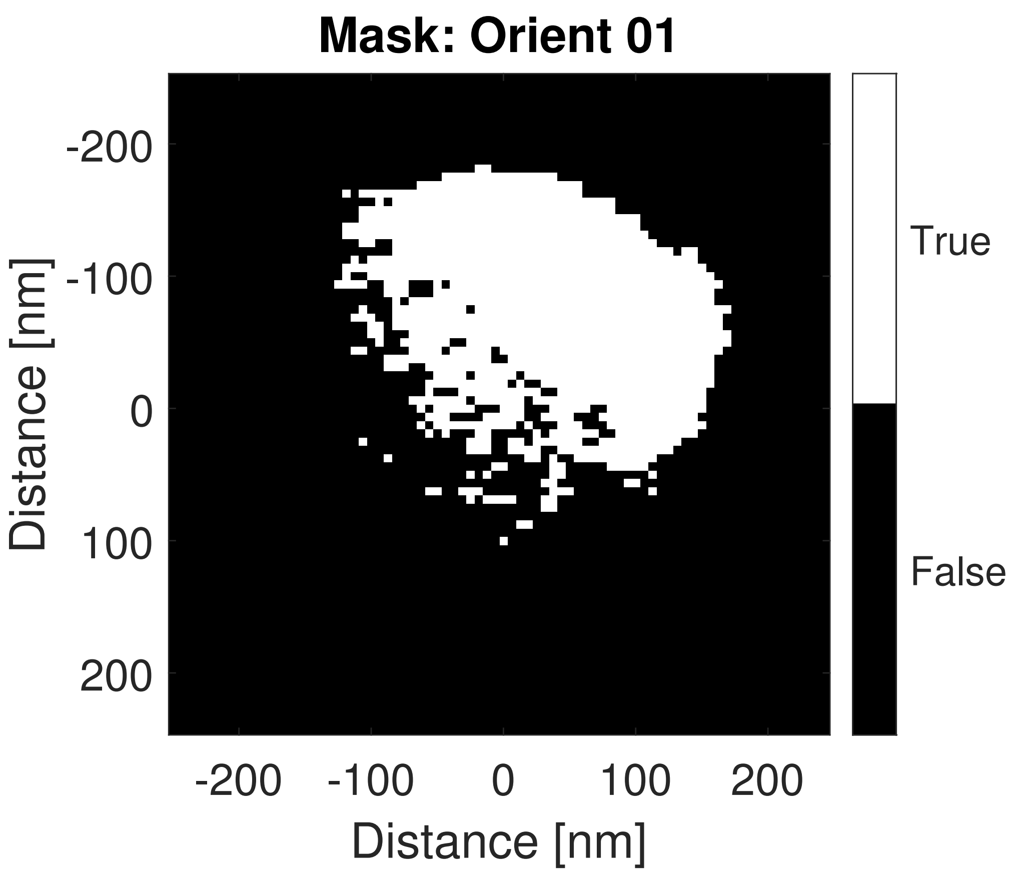


```
% Mask: Orient 02
% As above, the thresholding values were determined empirically and
% constrained to the "grain" mask
mask.orient2 = (vdf < 6000 & vdf > 4000) & mask.grain;

% Plot the mask for orientation 2 (larger mistilt)
figure('Color','w');
imagesc(x, x, mask.orient2);
set(gca, 'Colormap', gray(2));
colorbar;
set(findobj(gcf, 'Type', 'colorbar'), ...
    'Ticks', [0.25 0.75], 'TickLabels', {'False', 'True'}, ...
    'TickLength', 0);
axis image;
title('Mask: Orient 02')
set(gca, 'FontSize', 16);
xlabel('Distance [nm]');
ylabel('Distance [nm]');
```

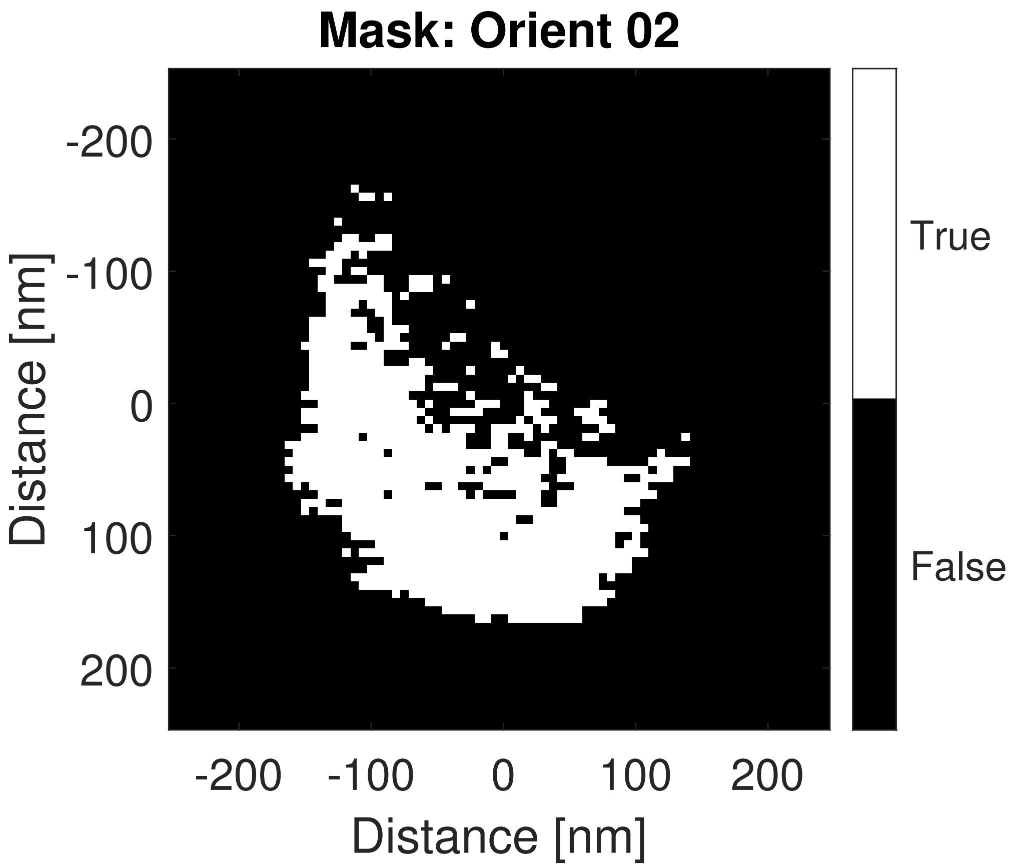

CBED patterns for different orientations

The average CBED patterns for the different orientation masks are generated here, reproducing part of the ROI figure from the paper.

```
% Compute the mean CBED pattern for orientation 1
CBED.orient1 = squeeze(mean(mean(permute(STEMDP, [3 4 1 2]) .* mask.orient1)));

% Plot the mean CBED pattern for orientation 1
figure('Color','w');
imagesc(qx, qx, CBED.orient1);
axis image;
colorbar;
title('Orientation 01')
set(gca, 'FontSize', 16);
xlabel('q_x [mrad]');
ylabel('q_y [mrad]');
set(gca, 'colormap', imGamma(gray(1000), 0.3));
```

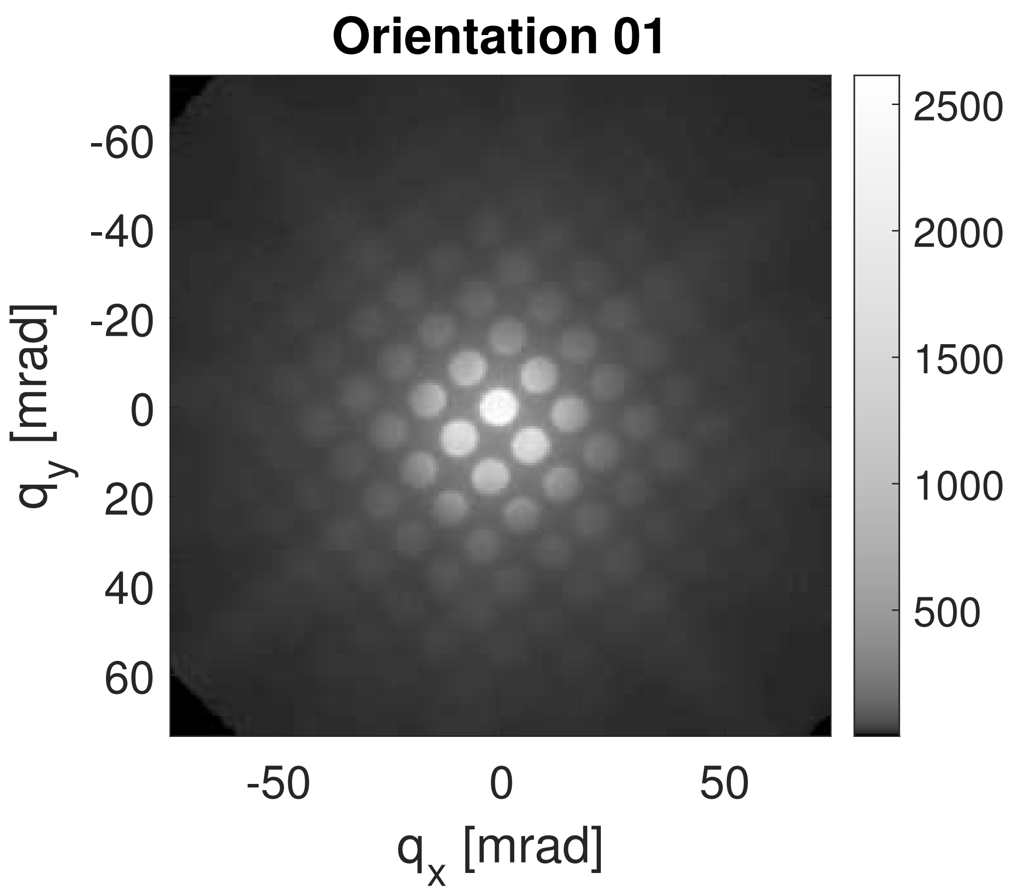


```
% Compute the mean CBED pattern for orientation 2
CBED.orient2 = squeeze(mean(mean(permute(STEMDP, [3 4 1 2]) .* mask.orient2)));

% Plot the mean CBED pattern for orientation 2
figure('Color','w');
imagesc(qx, qx, CBED.orient2);
axis image;
colorbar;
title('Orientation 02')
set(gca, 'FontSize', 16);
xlabel('q_x [mrad]');
ylabel('q_y [mrad]');
set(gca, 'colormap', imGamma(gray(1000), 0.3));
```

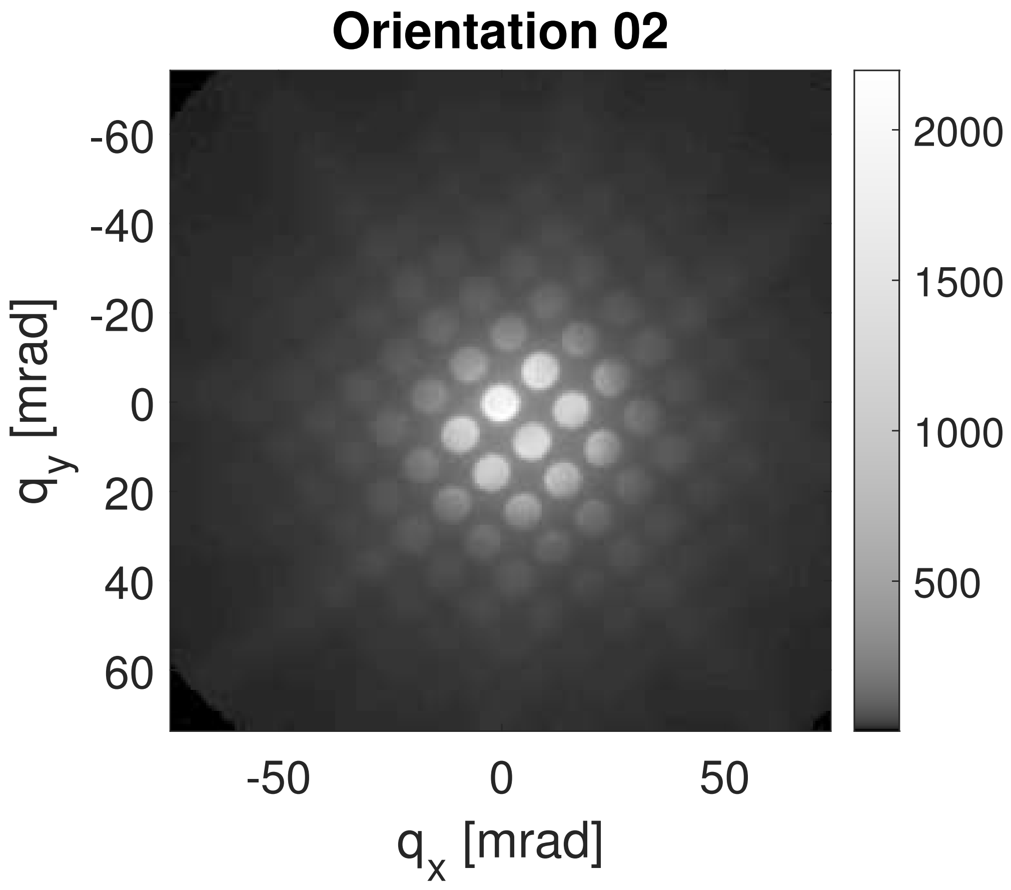


```
% Compute the mean CBED pattern for the entire grain
CBED.grain = squeeze(mean(mean(permute(STEMDP, [3 4 1 2]) .* mask.grain)));

% Plot the mean CBED pattern for the entire grain
figure('Color','w');
imagesc(qx, qx, CBED.grain);
axis image;
colorbar;
title('Mean grain orientation')
set(gca, 'FontSize', 16);
xlabel('q_x [mrad]');
ylabel('q_y [mrad]');
set(gca, 'colormap', imGamma(gray(1000), 0.3));
```

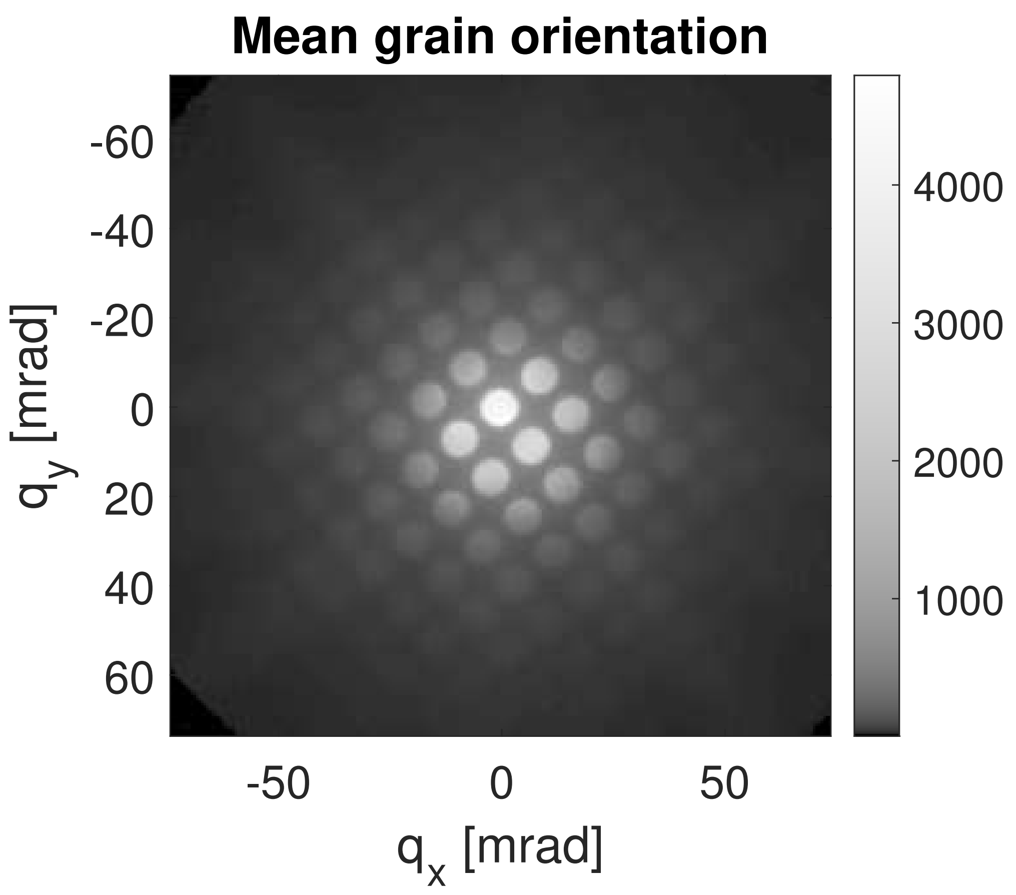

4D EELS pretreatment

The EELS pretreatment is performed before the stack of 2D EELS frames is assembled into a 4D datacube.

X-Ray spike identification and removal

In this section, x-ray spikes are identified and removed. The x-ray spikes are determined by looking for datapoints that deviate from a moving average by more than 5 sigma. The area surrounding these datapoints is then dilated and they are replaced by a linear interpolation from the nearest datapoints.

```
spikes = getXRaySpikeModel(A2, 'medWin', 17, ...
    'sigma', 5, 'plot', false, 'method', 'replace', 'dilate', 5);
A3 = applyXRaySpikeModel(A2, spikes);
```

Rough energy alignment (correcting for the drift-tube shift)

This first step corrects for the sawtooth drift tube offset that was applied during the data acquisition. Since the offset was acquired in a separate thread, the exact values are unknown. Cross-correlation with a reference spectrum is required to retrieve them.

The EELS data are first vertically summed (integration along q_y). The assumption here is that this is what happens under standard EELS acquisition. Also, each 2D EELS frame was acquired with the same drift tube offset, and this integration helps with the signal to noise ratio. Once this is done, a good looking spectrum that can be used as a reference is recorded to variable `ref`.

```
L = squeeze(sum(A3(1025:end, :, :), 2));
xL = 1:length(L);
ref = sum(L(:, 2960:2970), 2);
```

```
% Plot the reference spectrum
figure('color', 'w');
plot(ref, 'k');
xlabel('Channel number');
ylabel('Intensity');
set(gca, 'FontSize', 18);
title('Reference Spectrum');
```

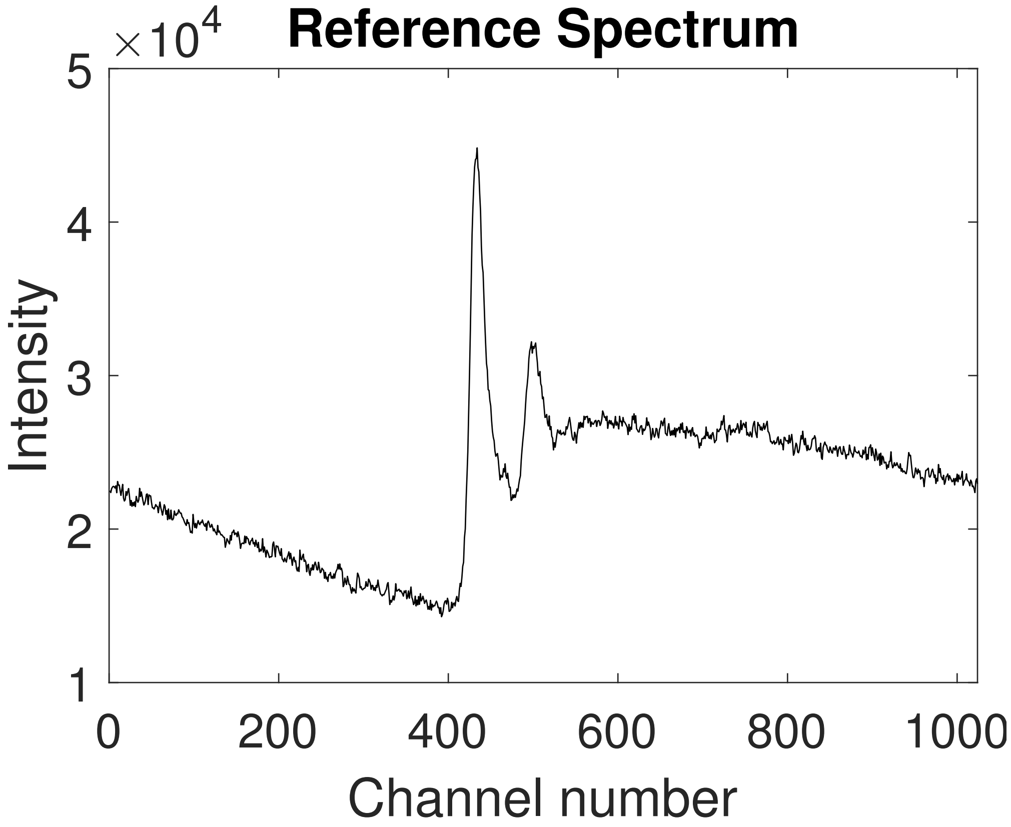

This step performs cross-correlation using the function `getISOmap`.

```
isomap = getISOmap(L, 'ref', ref, ...
    'diffOrder', 0, 'smooth', true, 'progressbar', false, 'interp', 10);
```

```
Getting isomap without parallel computing toolbox...Elapsed time is 5.083694 seconds.
```

Retrieval of the drift tube sawtooth function is inspected by plotting all of the shift values.

```
fig(1) = figure('color', 'w');
plot(xL, isomap.shift, 'k.', 'MarkerSize', 10);
xlabel('Scan index');
ylabel('Energy shift [channels]');
set(gca, 'fontsize', 18);
grid on;
title('Raw energy drift tube shift');
```

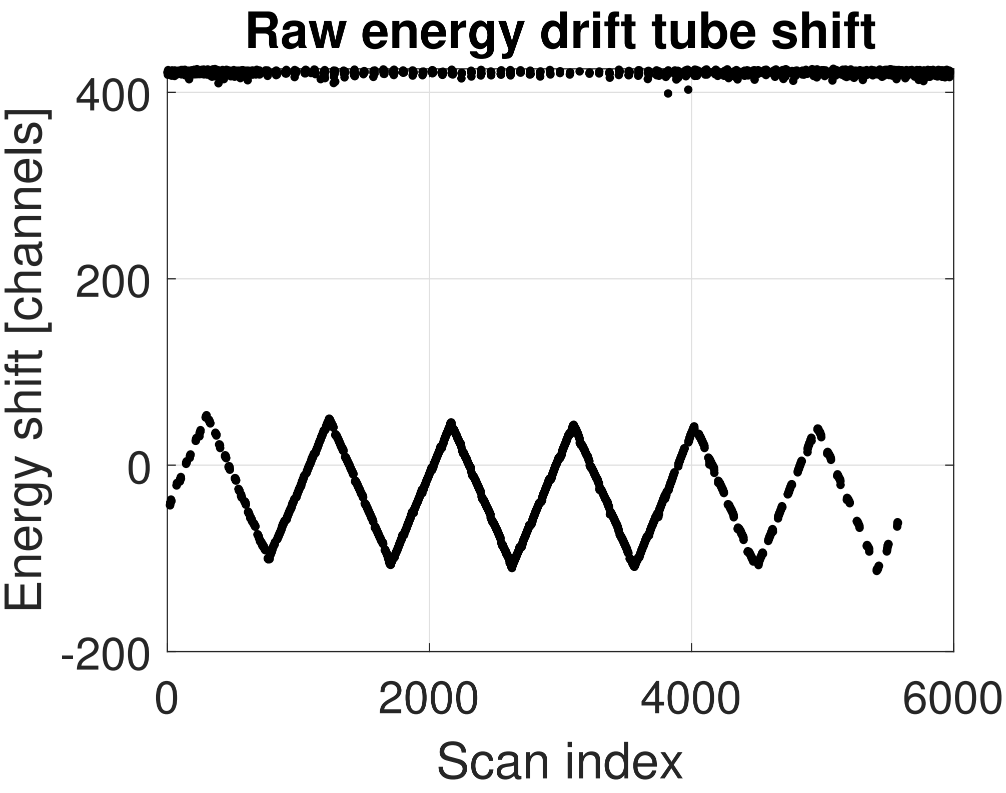

The sawtooth function is clearly visible. In regions where the Fe $L_{2,3}$ edges were too weak (such as over the background), the cross-correlation failed, giving a very large erroneous value.

Correct for outliers

Erroneous drift tube offset datapoints are easily corrected for by selecting extreme values. Extrapolation of the sawtooth function towards the end of the ESI acquisition is achieved by modelling the sawtooth function (although this is admittedly not necessary, as these datapoints don't contain iron and, thus, will not be used in the EMCD analysis).

```
badpx = isomap.shiftRaw > 80;
isomap.shift = filloutliers(isomap.shiftRaw, 'linear', 'OutlierLocations', badpx);
lastGoodPx = find(~badpx, 1, 'last');

% Manual estimation for pixels after the iron edge ends
isomap.shift(lastGoodPx + 1 : end) = 0.36.*xL(1 : length(L) - lastGoodPx) + isomap.shift(lastGoodPx);
isomap.shift(isomap.shift > max(isomap.shift(4300:lastGoodPx)) & xL > lastGoodPx) = ...
    max(isomap.shift(4300:lastGoodPx)) - (isomap.shift(isomap.shift > max(isomap.shift(4300:lastGoodPx)) & xL > lastGoodPx) ...
    - max(isomap.shift(4300:lastGoodPx)));

% Plot the correction
figure('color', 'w');
plot(xL, isomap.shiftRaw, 'k.', 'MarkerSize', 10, 'DisplayName', 'Raw Shift');
hold on;
plot(xL, isomap.shift, 'r', 'LineWidth', 1, 'DisplayName', 'Corrected Shift');
legend;
xlabel('Scan index');
```

```
ylabel('Energy shift [channels]');
set(gca, 'fontsize', 18);
grid on;
title('Raw energy drift tube shift');
```

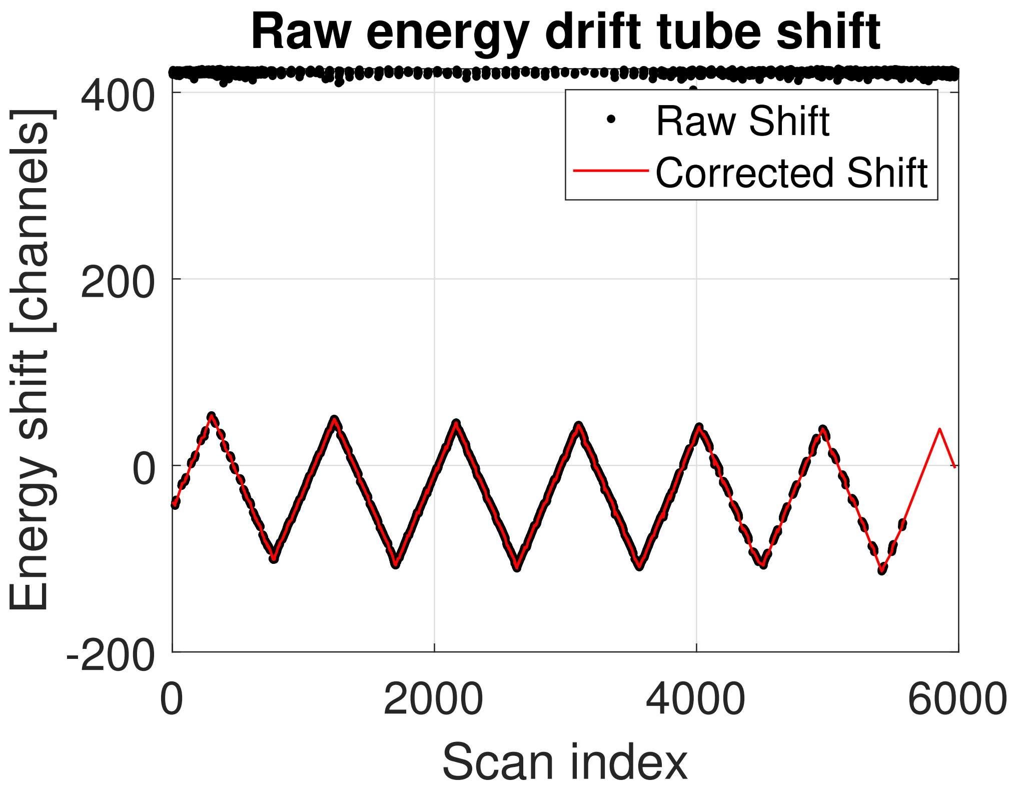

Apply the rough ISOmap and visualize it

The offset correction is now applied to the full 4D EELS datacube by iterating through each q_y vector.

```
for idx = 1:32
    [B(:, idx, :), cropMask, scanMask] = applyISOmap(A3(:, idx, :), isomap, '
        crop', false);
end

try
    sliceViewer(permute(B, [3 2 1]));
    colormap inferno(1024);
    axis normal
catch
    fprintf(1, 'Ensure that the GUI Layout Toolbox is installed');
end
```

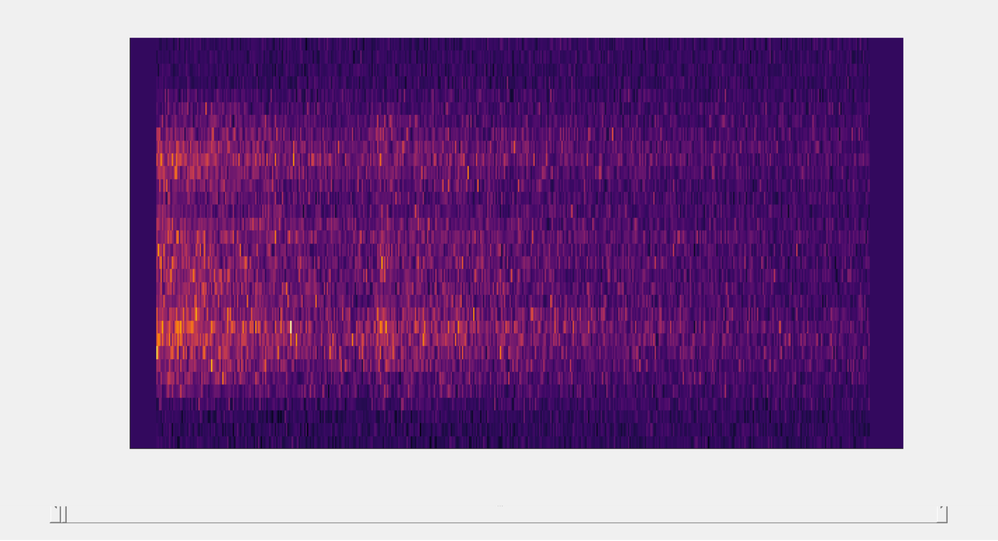

Fine energy drift correction

As discussed in the manuscript, the above energy drift correction only corrects (approximately) for the drift tube offset, providing gain averaging. However, close inspection of the EELS data reveals that there is some energy drift dependence on q_y as well. This is corrected for here by using a peak shifting methodology based on regressing the spectral mean and its first derivation against the raw data. The input for this step is the roughly-aligned dataset.

```
mask.Ref = 1500:1615; %channels.  Only select Fe L3
mask.meanEELSSpectra = mean(B, 1); % Restrict to iron grain
mask.meanEELSSpectra = squeeze(mask.meanEELSSpectra);
mask.BG = mask.meanEELSSpectra < 28; %Based on 4D summation

% taylorShift is designed for typical 3D hyperspectral EELS datacubes, which
% we currently have, since we have not interpolated to an (x, y) datacube.
[~, shifts] = taylorShift(make2D(B(mask.Ref, :, :)), 'nIter', 5, 'mask', ~mask.
    BG(:));
```

```
Iteration number 01
Iteration number 02
Iteration number 03
Iteration number 04
Iteration number 05
```

The energy offset of Fe L_3 as a function of both scan index (pixel position) and q_y can now be visualized. A noticable energy shift between the middle region (low values of q_y) and the outer regions is observed. Thus, this correction step is crucial when comparing chiral spectra that are derived from q_y regions of opposite sign.

```
% Plot the qy - Npx energy offset map
figure('color', 'w');
imagesc(1:length(L), qy, reshape(sum(shifts, 2), 32, length(L)));
axis normal;
colorbar;
set(gca, 'Colormap', flipud(brewermap(1024, 'PuOr')));
set(gca, 'CLim', 10 .* [-1 1]); % Center around zero
set(gca, 'FontSize', 18);
xlabel('Scan Index');
ylabel('q_y [mrad]');
title('q-dependent energy offset');
```

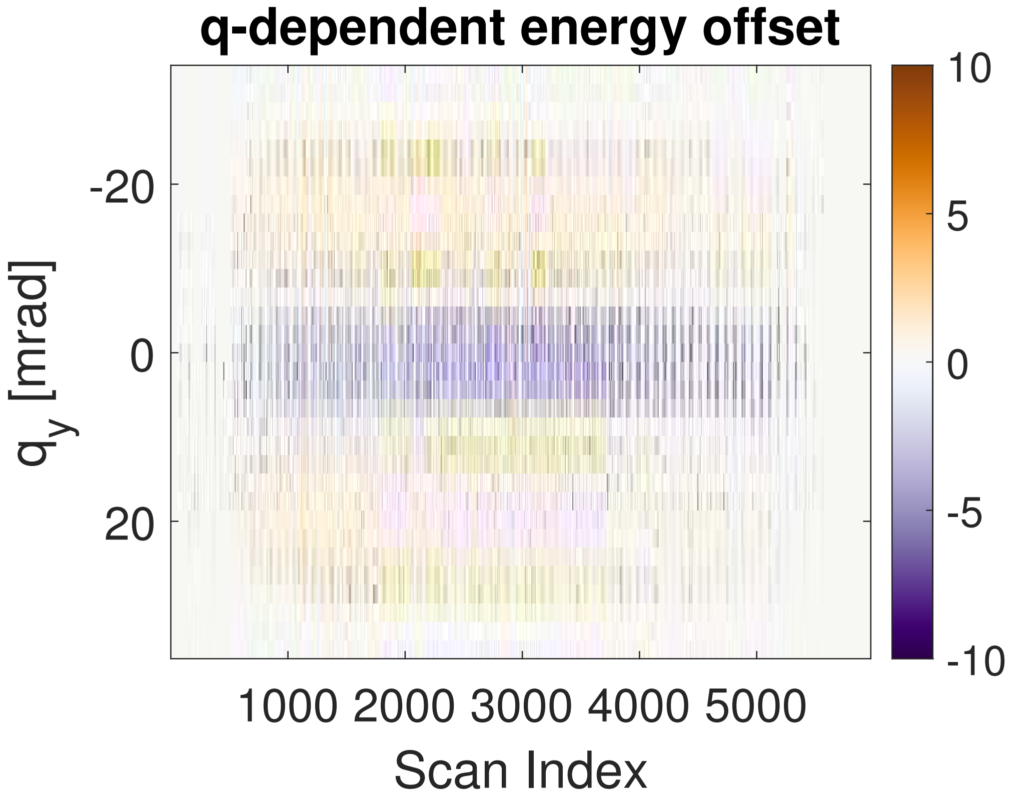

This energy offset map can now be added the drift tube offset from the previous section. This is final energy offset map is applied to the raw data.

```
% Drift tube offset
isomapFinal = isomap;

% Expand to qy x L dimensions
isomapFinal.shift = repmat(isomap.shift, [32 1]);

% Add the q-dependence to the drift tube offset
isomapFinal.shift = isomapFinal.shift + reshape(sum(shifts, 2), 32, length(L));

% Plot the result for inspection
figure('Color','w');
imagesc(1:length(L), qy, isomapFinal.shift);
axis normal;
colorbar;
set(gca, 'Colormap', flipud(brewermap(1024, 'PuOr')));
set(gca, 'CLim', max(abs(get(gca, 'CLim'))) .* [-1 1]); % Center around zero
set(gca, 'FontSize', 18);
xlabel('Scan Index');
ylabel('q_y [mrad]');
title('Final energy drift correction');
```

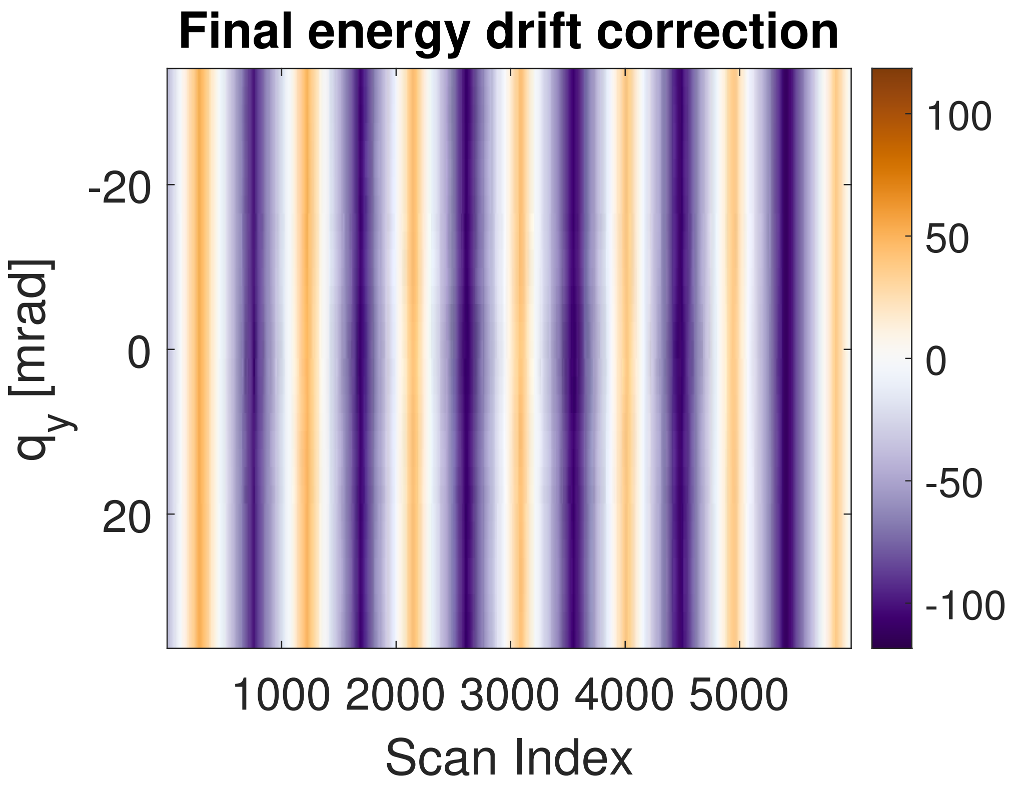

Apply the complete energy drift offset

We now return to the original (raw) dataset and apply the full energy drift correction. This includes both the drift tube offset as well as the q_y-dependent correction. The result is saved in variable `C`, which is ready for interpolation into a 4D datacube (allowing it to be correlated to the STEM-DP datacube through the x and y spatial dimensions).

```
C = applyISOmap(A3, isomapFinal, 'crop', true);

% Interactive visualization
try
    viewESI(1:size(C, 1), 1, C); axis normal; colormap inferno(1024);
catch
    fprintf(1, 'Ensure that the GUI Layout Toolbox is installed');
end
```

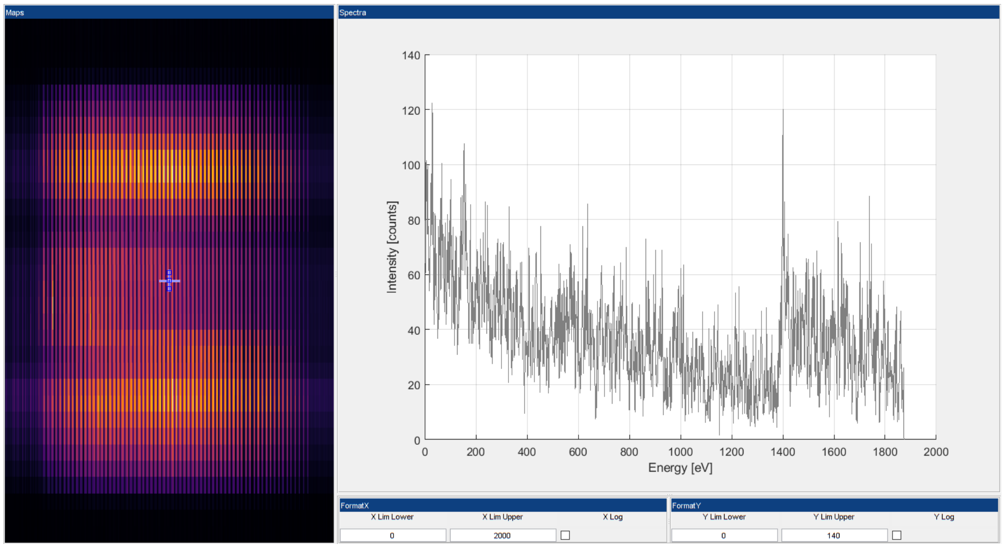

A new energy vector is generated at this stage. Channel 1401, which is the maximum of the Fe L_3 edge, is defined to be 709 eV. The new energy vector is saved as variable `e`.

```
e = (((0:size(C, 1)-1) - 1401).*0.2 + 709)';
```

Interpolation to 4D datacube

For the final preprocessing stage, the stack of 2D EELS frames (dimensions $\Delta E \times q_y \times N_{px}$) is assembled via interpolation into a 4D datacube (dimensions $\Delta E \times q_y \times x \times y$). This datacube is moreover cropped to the Fe $L_{2,3}$ edges and extreme values of q_y falling outside of the passthrough region of the aperture are removed to cut down on the data size.

```
goodQ = 5:28; % qy.  Selects qy values that reside within the aperture holes
mskE = 1000:1800; % channels.  Selects the iron edges
nRows = 80; % Number of rows (same as STEM-DP)
nCols = 80; % Number of columns (same as STEM-DP)
qy2 = qy(goodQ); % Crop the qy vector
clear temp temp2 maphistogram

% Preallocate the final 4D EELS variable
DPEELS = zeros(length(mskE), length(goodQ), nCols, nRows);
```

To write the 2D EELS frames into a 4D datacube, the following `for` loop iterates through each row. The timestamps (in variable `ts`) assign the frame index to the appropriate row. While most probe positions can be assigned a single 2D EELS frame, some will be missing and some will have excess frames. This is mitigated through the use of a nearest neighbor interpolation for each row, which is what the code below achieves.

```
for idx = 1:(nRows-1)
    temp = C(mskE, goodQ, ts.idxNewRow(idx):(ts.idxNewRow(idx+1)-1));
    temp2 = resampleESI(temp, [length(mskE) length(goodQ) nCols], 'nearest');
    DPEELS(:, :, ts.finalrowindex(idx):ts.finalrowindex(idx+1)-1) = temp2;
    maphistogram(:, idx) = ...
        histcounts(ts.activeframes(ts.idxNewRow(idx) : (ts.idxNewRow(idx+1)-1)), ...
        [ts.pxbegin(ts.finalrowindex(idx) : ts.finalrowindex(idx+1)-1); ts.pxend(ts.finalrowindex(idx+1)-1)]);
end
```

The 4D EELS datacube is now reshaped into the four dimensions and permuted for convenience.

```
DPEELS = reshape(DPEELS, length(mskE), length(goodQ), nCols, nRows);
DPEELS = permute(DPEELS, [2 1 4 3]);
```

A comparison with the virtual HAADF image from the CBED datacube can be used to ensure that the spatial registration is reasonable. This is achieved in the 4D EELS datacube here by integrating along both the ΔE and q_y dimensions, yielding an x, y map that represents the total differential inelastic scattering cross sections.

```
% Plot 4D EELS map
figure('Color','w');
imagesc(x, x, squeeze(sum(sum(DPEELS, 2), 1)));
colormap gray;
axis image;
colorbar;
title('4D EELS interpolation')
set(gca, 'FontSize', 16);
xlabel('Distance [nm]');
ylabel('Distance [nm]');
```

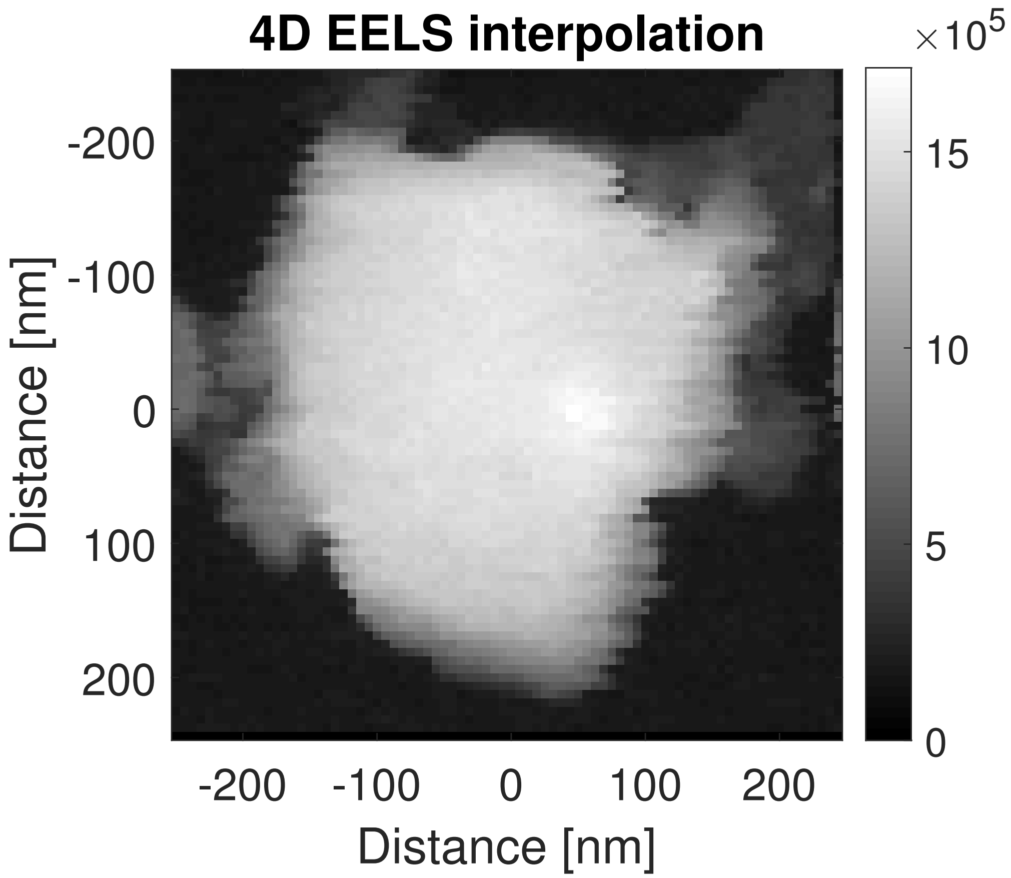

The number of 2D EELS frames acquired at each x, y position are checked by inspecting the map histogram, as previously done for the STEM-DP datacube.

```
% Generate the 4D EELS histogram
maphistogram = maphistogram'; % Transpose x, y
```

```matlab

% Plot the 4D EELS histogram
figure('Color','w');
imagesc(x, x, maphistogram);
set(gca, 'colormap', gray(max(maphistogram(:))+1));
axis image;
colorbar;
title('2D EELS frames per pixel')
set(gca, 'FontSize', 16);
xlabel('Distance [nm]');
ylabel('Distance [nm]');
set(findobj(gcf, 'Type', 'colorbar'), 'Ticks', [0:max(maphistogram(:))]);
```

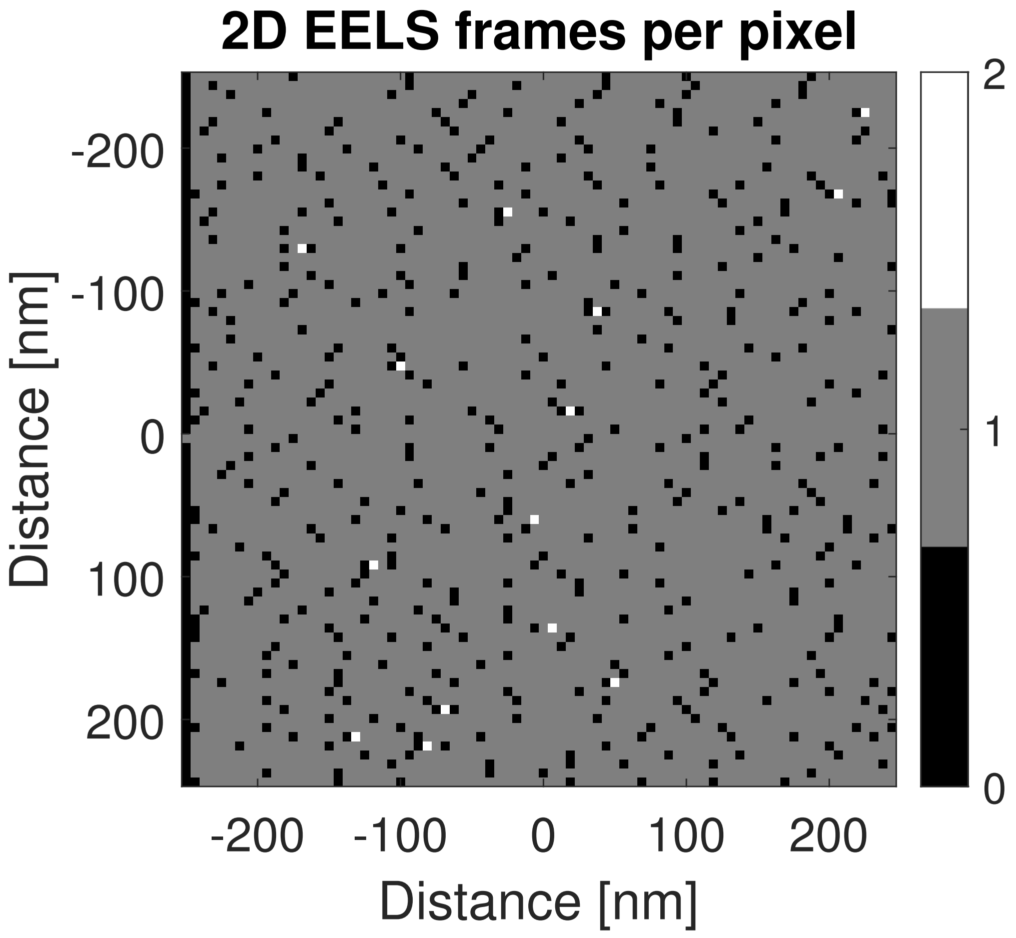

This marks the end of the pretreatment stage for the 4D EELS dataset.

Candidate EMCD signal extraction:

Necessary function handles

`q` is a simple function handle that selects the opposing q-values for the summed 2D EELS spectra. This assumes that the center of the summation lies at `q = 0`. This was determined by careful placement of the patterned aperture during experimental acquisition, and the centering we belive to be most accurate is applied here. If you want to see the effect of changing this center value, then re-run the section above with different values for `goodQ`.

```matlab
q = @(S, ind) [sum(S(ind, :), 1); sum(S(end-ind+1, :), 1)]';
```

`sp` is a function handle that sharpens (profile matches) one of the two chiral spectra. The approach is a standard removal of the second derivative. Validation of this approach can be performed by integrating the second derivative. If there is a zero-crossing between the L_3 and L_2 peaks, then the area under the peaks will not change and, hence, this will not directly bias the calculation of m_L/m_S (although this is not explicitly studied in this manuscript due to the low SNR). This profile matching step is necessary since there is a very slight energy blur that arises from spectrometer errors. The matching routine is parameterized by multiplying the second derivative by a scalar value (`k`) and this is what is allowed to vary in the optimization code for EMCD signal extraction. This function handle serves to set an initial starting parameter, which is occasionally needed to avoid reaching a local minimum. This is something that you can play around with to see the result to get a feel for what it does to the data. The starting paremeter used below was empirically determined, but typically was in the range of -1 to -2.

```
sp = @(Sp, k) Sp - k.*gradient(gradient(smooth(Sp)));
```

To ensure that the `sp` function does not change the area under the curve:

```
DPEELSsum.all = sum(sum(DPEELS.*permute(mask.grain, [4 3 1 2]), 4), 3);
testSharpening = q(DPEELSsum.all, 1:12);
testSharpening(:, 1) = []; % Remove chiral plus

% Get new energy vector
e3 = ((1:size(testSharpening, 1))-find(testSharpening(:, 1) == max(testSharpening(:, 1)))).*0.2 + 710;
e3 = e3(:);

% Make the figure
figure('color', 'w');
subplot(3, 1, 1);
% Plot the raw spectrum
plot(e3, testSharpening, 'k', 'LineWidth', 2);
title('Raw spectrum');
subplot(3, 1, 2);
% Plot the second derivative, scaled by -1 (causes broadening).  Smoothed
% first to reduce the noise contribution
plot(e3, -1*gradient(gradient(smooth(testSharpening))), 'k', ...
    'LineWidth', 2);
title('Second derivative');
subplot(3, 1, 3);
% Plot the cumulative sum of the second derivative (which is basically the
% first derivative).  The values between Fe L3 and L2 are close to 0;
% hence, the area under the curves does not change significantly.
plot(e3, cumsum(-1*gradient(gradient(smooth(testSharpening)))), 'k', ...
    'LineWidth', 2);
title('Cumulative sum of second derivative');
set(findall(gcf, 'type', 'axes'), 'xlim', [695 745], 'FontSize', 12, ...
    'XGrid', 'on', 'YGrid', 'on');
xlabel('Energy [eV]');
set(gcf, 'Position', [1 1 1 2] .* get(gcf, 'Position'));
```

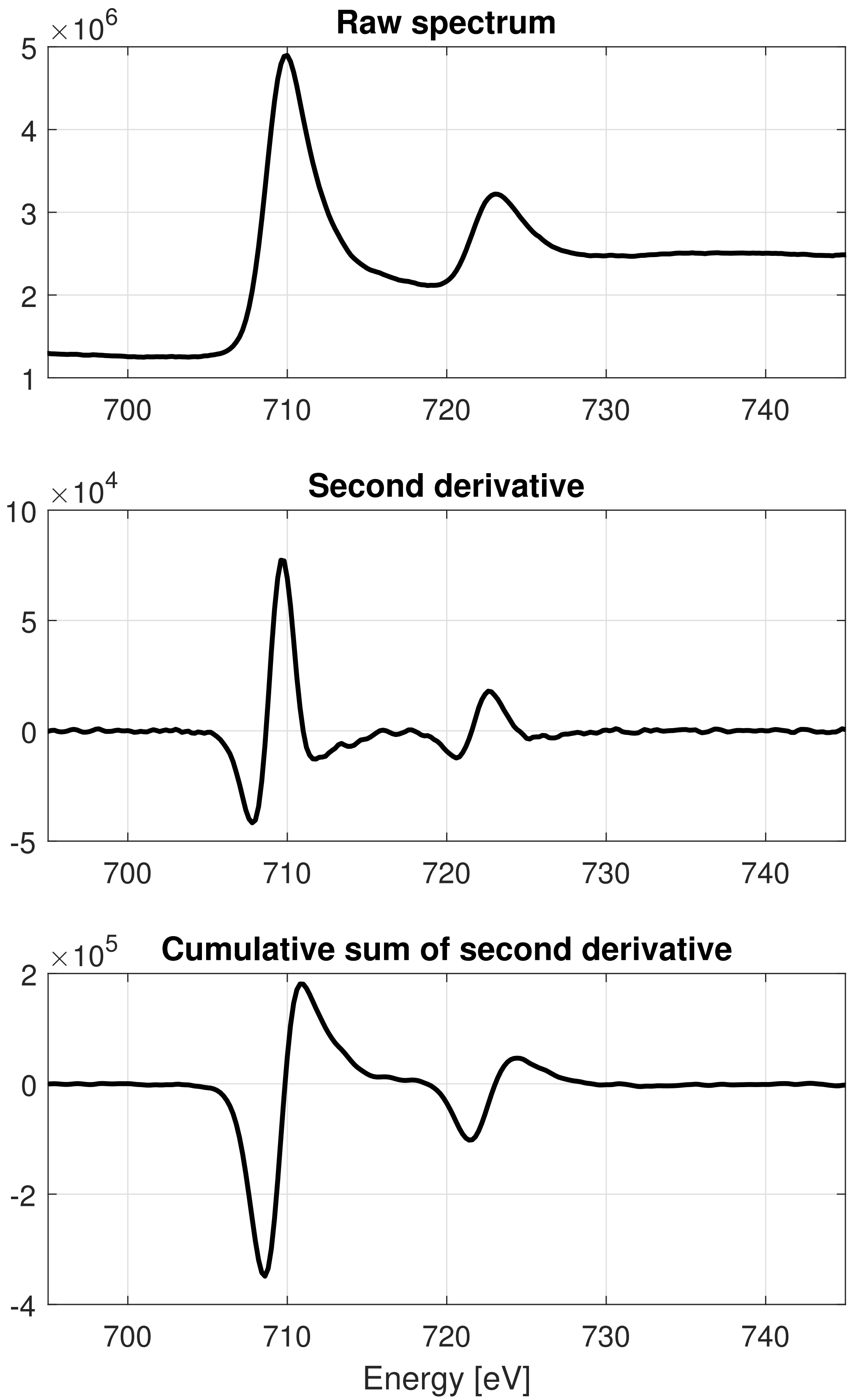

Introduction to the `optimMLMS.m` function

The following sections run the optimization function `optimMLMS.m` for EMCD signal extraction. This function is outlined in the manuscript but more details are available within the function code itself. In short, it takes two chiral

spectra as input (output from `q` above), determines starting parameters (`prepEMCDoptim.m`), writes these to variable `fi` (only accessible in the function workspace), passes this to Matlab's `fmincon` function along with the objective function `objMLMS.m`. The parameters in `fi` are listed in the table in the manuscript. Some of these parameters are used to generate a fit function to the EMCD signal (which is the difference between background-subtracted, profile-matched, properly normalized chiral spectra). This fit function is subtracted from the raw EMCD signal leaving residual errors. The sum of the square of these errors is minimized with `fmincon`. The resulting optimal parameters are returned as variable `fo` and written to the active Matlab workspace. A struct containing all of these parameters as well as the processed data is returned as `fStruct` in the Matlab workspace and can then be inspected.

As input parameters, a number of different models can be used. The standard inverse power law and the linear combination of power laws (Cornell Spectrum Imager) are options for the pre-edge background model. The post-edge linearization can either use the standard integration approach or the linear regression approach discussed in the manuscript. The residual errors that are minimized can be weighted (not performed here) and decoupled as needed. In this case, the pre-edge regions for both chiral spectra are "decoupled" and optimized in serial with the residuals from the EMCD signal, which helps keep the backgrounds more reasonable. Finally, the EMCD signal model can be chosen. The standard double-gaussian approach can be chosen, but this fails to capture the extended tails of the EMCD signal. The pseudo-Voigt function approach is called with the `'GLmix'` input argument and is what is performed here. An additional skewed Gaussian model is available for playing with. Details are provided within the function itself.

The data are visualized by calling the function `plotEMCDfitCum.m`, which accepts the struct output from `optimMLMS.m` as an argument and returns a formatted plot that is used in the manuscript. Unfortunately, at the time of writing, Matlab's Live Script environment does not allow for interactive or updatable figures. Consequently, it is not possible to watch the progression of this optimization routine during the iterations. This can be accomplished by copying the code below that calls `optimMLMS` (along with all its additional arguments) but adding a fourth output argument (for example, `flm`) and executing this in the Matlab command line directly. This will both allow the graph to update at every iteration, but will also save each frame (iteration) as an entry in a large struct variable in the workspace (`flm`). Then, the included `makeMovie.m` function can be called as follows:

```
makeMovie(flm, 'FileName.mp4', 10);
```

This will create and save an `.mp4` file into the current path with the name `'FileName.mp4'` and, in this case, a speed of 10 frames per second.

Using `mask.orient01` (close to zone axis) and restricted q_y

The first EMCD signal is extracted from the sample region where the iron grain was oriented close to a zone axis. This is the ROI called "Orient01" in the paper. The values of q_y that are used for this signal extraction are limited to the "outer" q_y region. The reason for this is clear from below when the q_y maps are generated.

```
win.pre = [650 700];
win.post = [730 745];

% Get the spectra from the "outer" four rows of qy
DPEELSsum.orient1 = sum(sum(DPEELS.*permute(mask.orient1, [4 3 1 2]), 4), 3);
tmp = q(DPEELSsum.orient1, 1:4);

% Make a new energy vector
e3 = ((1:size(tmp, 1))-find(tmp(:, 1) == max(tmp(:, 1)))).*0.2 + 710;
e3 = e3(:);

% Run the optimization function
[fo, mLmS, fStruct] = optimMLMS(e3, tmp(:, 1), shift1D(sp(tmp(:, 2), -1.5), 0,
    true), win, ...
     'preModel', 'Power Law', ...
```

```
    'postModel', 'Linear', ...
    'SScalculation', 'Decoupled', ...
    'sigModel', 'GLmix', ...
    'xlim', [695 745], ...
    'deblur', true, ...
    'shift', true, ...
    'algorithm', 'active-set');

% Plot the result
plotEMCDfitCum(fStruct, 'xlim', [695 745]);
```

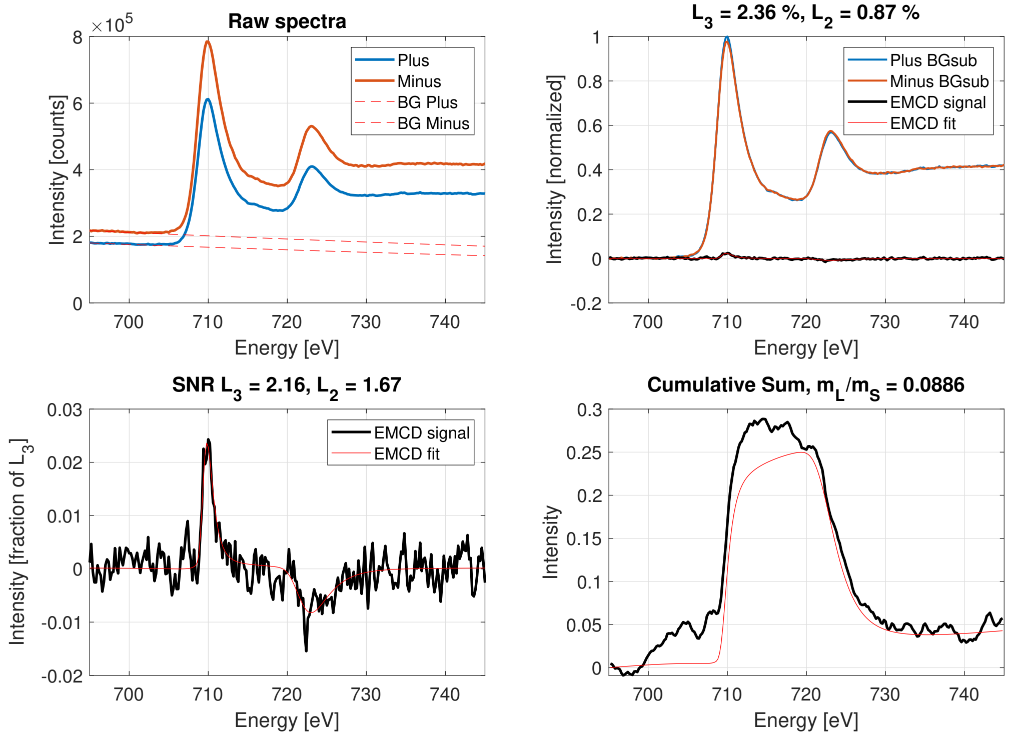

This reproduces figure 5 in the manuscript.

Using `mask.orient02` (tilted away from zone axis) and restricted q_y

The second EMCD signal is extracted from the sample region where the iron grain was mistilted. This is the ROI called "Orient02" in the paper. The values of q_y are the same as before, in order to compare the effect of crystallographic orientation on the EMCD signal quality

```
win.pre = [650 700];
win.post = [730 745];

% Get the spectra from the "outer" four rows of qy
DPEELSsum.orient2 = sum(sum(DPEELS.*permute(mask.orient2, [4 3 1 2]), 4), 3);
tmp = q(DPEELSsum.orient2, 1:4);

% Make a new energy vector
e3 = ((1:size(tmp, 1))-find(tmp(:, 1) == max(tmp(:, 1)))).*0.2 + 710;
e3 = e3(:);

% Run the optimization function
```

```
[fo, mLmS, fStruct] = optimMLMS(e3, tmp(:, 1), shift1D(sp(tmp(:, 2), -1.5), 0,
    true), win, ...
    'preModel', 'Power Law', ...
    'postModel', 'Linear', ...
    'SScalculation', 'Decoupled', ...
    'sigModel', 'GLmix', ...
    'xlim', [695 745], ...
    'deblur', true, ...
    'shift', true, ...
    'algorithm', 'active-set');

% Plot the result
plotEMCDfitCum(fStruct, 'xlim', [695 745]);
```

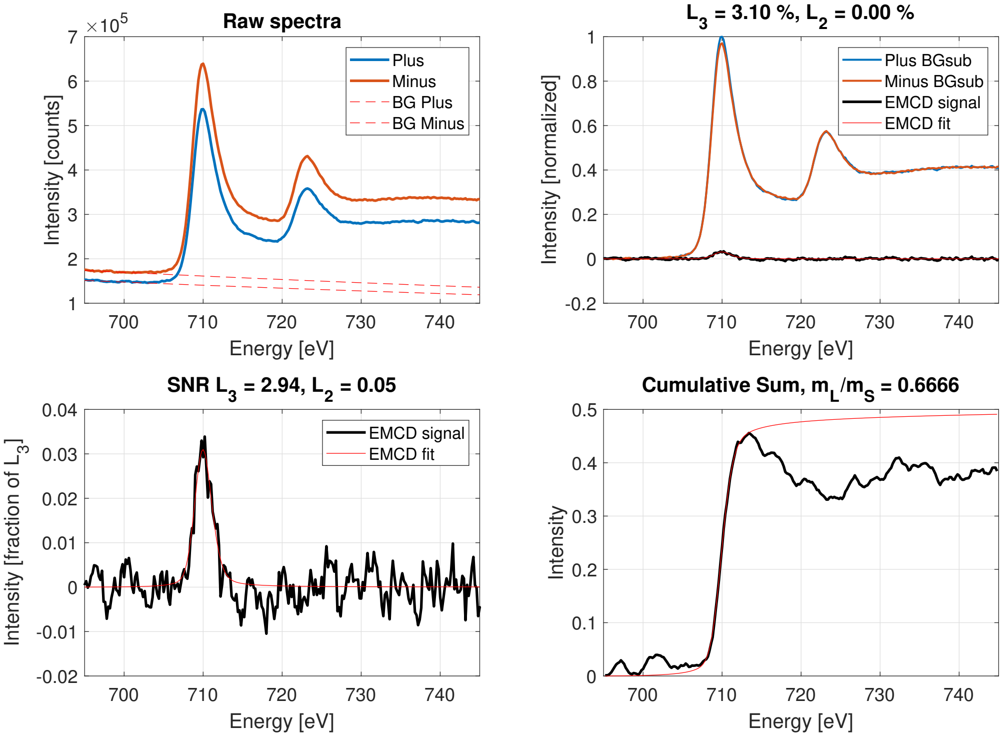

This reproduces figure 6 in the manuscript.

Using `mask.grain` and all q_y values

The third EMCD signal is a summation over all crystallographic orientations and q_y values.

```
win.pre = [650 700];
win.post = [730 745];

% Get the spectra from all qy values using the "grain" mask
DPEELSsum.all = sum(sum(DPEELS.*permute(mask.grain, [4 3 1 2]), 4), 3);
tmp = q(DPEELSsum.all, 1:12);

% Make a new energy vector
e3 = ((1:size(tmp, 1))-find(tmp(:, 1) == max(tmp(:, 1)))).*0.2 + 710;
e3 = e3(:);

```

```
% Run the optimization function
[fo, mLmS, fStruct] = optimMLMS(e3, tmp(:, 1), shift1D(sp(tmp(:, 2), -1.5), 0,
    true), win, ...
     'preModel', 'Power Law', ...
     'postModel', 'Linear', ...
     'SScalculation', 'Decoupled', ...
     'sigModel', 'GLmix', ...
     'xlim', [695 745], ...
     'deblur', true, ...
     'shift', true, ...
     'algorithm', 'active-set');

% Plot the result
plotEMCDfitCum(fStruct, 'xlim', [695 745]);
```

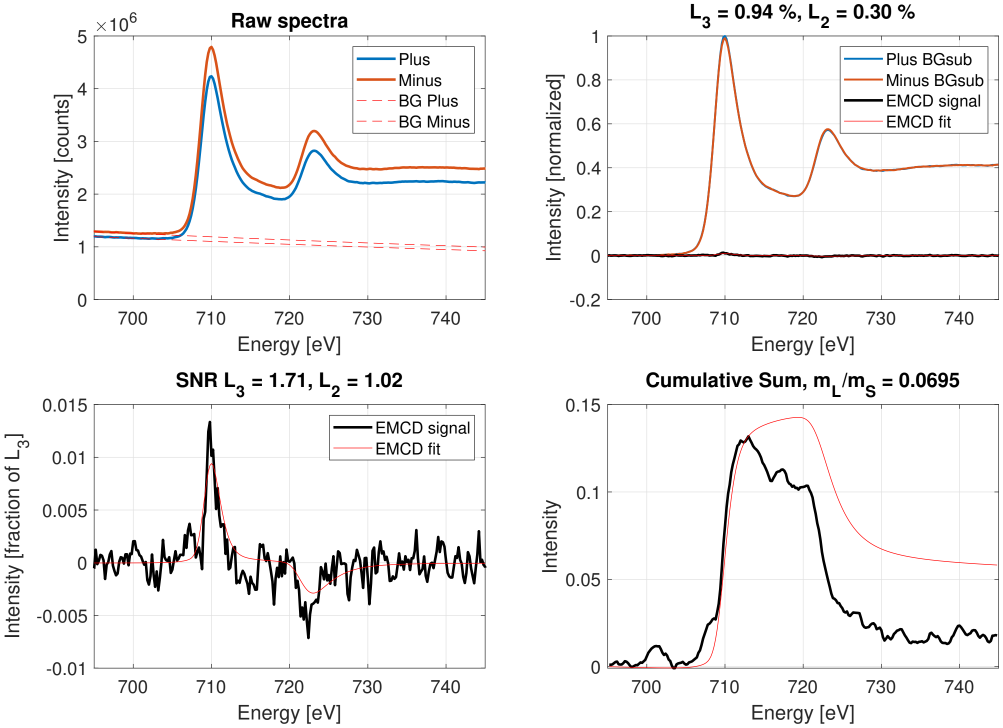

This reproduces figure 7 in the manuscript.

***Using* `mask.grain` *(integral over all orientations) and restricted* q_y**

The effect of q_y can be assessed by integrating over a restricted range of q_y values. This EMCD signal is extracted using the "outer" 4 rows, as with the orientation masks above.

```
win.pre = [650 700];
win.post = [730 745];

% Get the spectra from "outer" qy values using the "grain" mask
DPEELSsum.grain = sum(sum(DPEELS.*permute(mask.grain, [4 3 1 2]), 4), 3);
tmp = q(DPEELSsum.grain, 1:4);

% Make a new energy vector
e3 = ((1:size(tmp, 1))-find(tmp(:, 1) == max(tmp(:, 1)))).*0.2 + 710;
```

```
e3 = e3(:);

% Run the optimization function
[fo, mLmS, fStruct] = optimMLMS(e3, tmp(:, 1), shift1D(sp(tmp(:, 2), -1.5), 0,
    true), win, ...
     'preModel', 'Power Law', ...
     'postModel', 'Linear', ...
     'SScalculation', 'Decoupled', ...
     'sigModel', 'GLmix', ...
     'xlim', [695 745], ...
     'deblur', true, ...
     'shift', true, ...
     'algorithm', 'active-set');

% Plot the result
plotEMCDfitCum(fStruct, 'xlim', [695 745]);
```

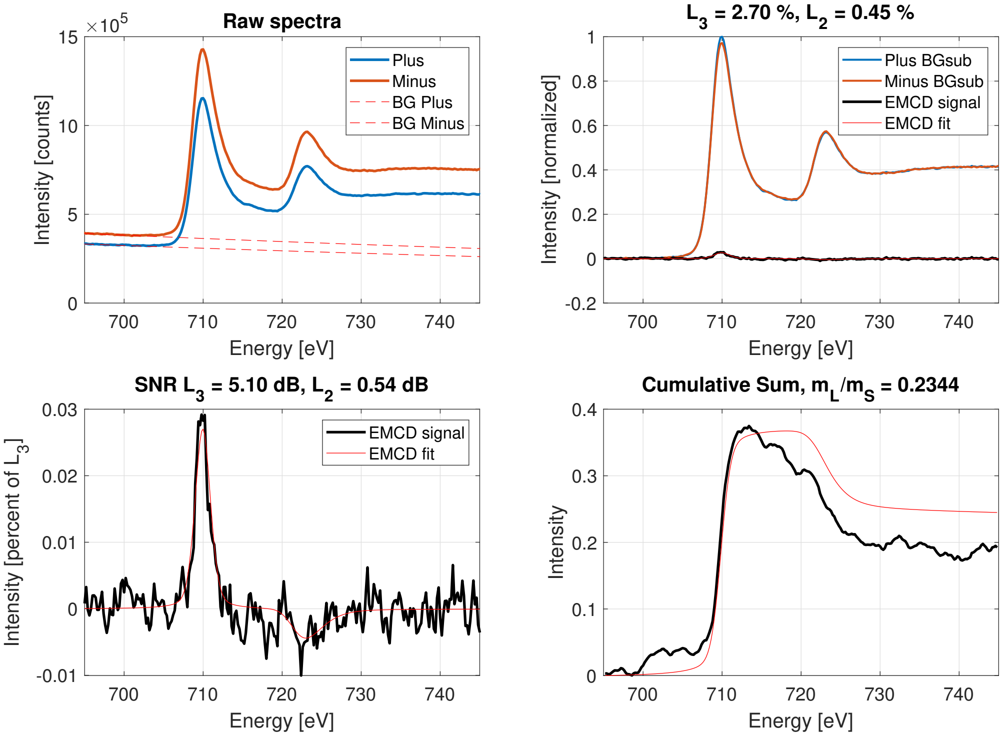

This reproduces figure 8 in the manuscript.

EMCD q-maps

The $q-E$ maps for the EMCD signal can be created by running `optimMLMS.m` on each individual q_y value. This is achieved by iterating over all 12 available conjugate q_y vectors in the dataset. All three of the ROI masks are tested with this approach.

Line profile for `mask.orient1`

This is the map for the "Orient01" mask

```
win.pre = [650 700];
win.post = [730 745];
```

```

clear flm fo fStruct;
tic;
for idx = 12:-1:1
    fprintf(1, '\nIteration %02d', idx);
    tmp = q(DPEELSsum.orient1, idx);
    [fo(idx, :), mLmS(idx), fStruct(idx)] = optimMLMS(e3, ...
        tmp(:, 1), shift1D(sp(tmp(:, 2), -1.5), 0, true), win, ...
        'preModel', 'Power Law', ...
        'postModel', 'Linear', ...
        'SScalculation', 'Decoupled', ...
        'sigModel', 'GLmix', ...
        'xlim', [695 745], ...
        'deblur', true, ...
        'shift', true, ...
        'algorithm', 'active-set');
    close(gcf);
end
```

```
Iteration 12
Iteration 11
Iteration 10
Iteration 09
Iteration 08
Iteration 07
Iteration 06
Iteration 05
Iteration 04
Iteration 03
Iteration 02
Iteration 01
```

```
toc;
```

```
Elapsed time is 27.441344 seconds.
```

```
% Plot the Orient01 q-E map
LineEELS.orient1 = fStruct;
figure('Color', 'w');
imagesc(e3, abs(qy2(1:12)), [LineEELS.orient1.EMCD]');
pbaspect([5 1 1]);
set(gca, 'YDir', 'normal');
set(gca, 'colormap', flipud(brewermap(1000, 'PuOr')));
set(gca, 'clim', [-0.04 0.04], 'xlim', [695 745]);
ylabel('q_y [mrad]');
xlabel('Energy [eV]');
title('q_y map for orientation 01 mask')
```

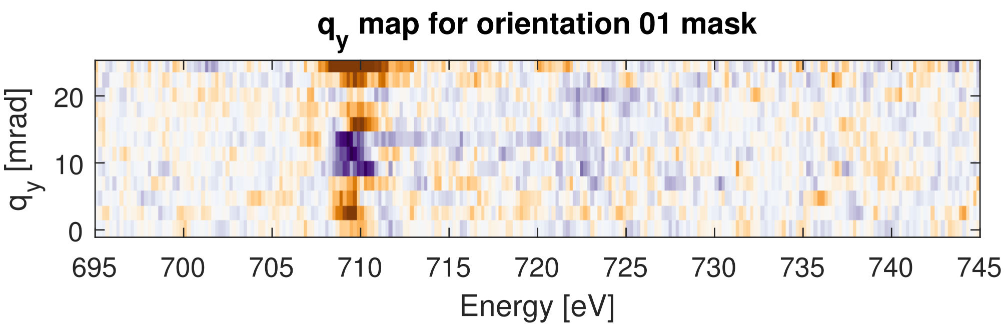

Line profile for `mask.orient2`

This is the map for the "Orient02" mask

```
win.pre = [650 700];
win.post = [730 745];

clear flm fo fStruct;
tic;
for idx = 12:-1:1
    fprintf(1, '\nIteration %02d', idx);
    tmp = q(DPEELSsum.orient2, idx);
    [fo(idx, :), mLmS(idx), fStruct(idx)] = optimMLMS(e3, ...
        tmp(:, 1), shift1D(sp(tmp(:, 2), -1.5), 0, true), win, ...
        'preModel', 'Power Law', ...
        'postModel', 'Linear', ...
        'SScalculation', 'Decoupled', ...
        'sigModel', 'GLmix', ...
        'xlim', [695 745], ...
        'deblur', true, ...
        'shift', true, ...
        'algorithm', 'active-set');
    close(gcf);
end
```

```
Iteration 12
Iteration 11
Iteration 10
Iteration 09
Iteration 08
Iteration 07
Iteration 06
Iteration 05
Iteration 04
Iteration 03
Iteration 02
Iteration 01
```

```
toc;
```

```
Elapsed time is 21.993646 seconds.
```

```
% Plot the Orient02 q-E map
LineEELS.orient2 = fStruct;
figure('Color', 'w');
imagesc(e3, abs(qy2(1:12)), [LineEELS.orient2.EMCD]');
```

```
pbaspect([5 1 1]);
set(gca, 'YDir', 'normal');
set(gca, 'colormap', flipud(brewermap(1000, 'PuOr')));
set(gca, 'clim', [-0.04 0.04], 'xlim', [695 745]);
ylabel('q_y [mrad]');
xlabel('Energy [eV]');
title('q_y map for orientation 02 mask')
```

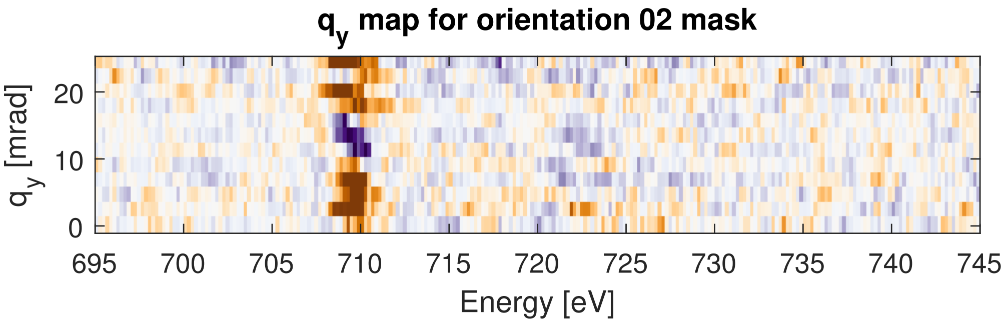

Line profile for `mask.grain`

This is the map for the full grain mask

```
win.pre = [650 700];
win.post = [730 745];

clear flm fo fStruct;
tic;
for idx = 12:-1:1
    fprintf(1, '\nIteration %02d', idx);
    tmp = q(DPEELSsum.grain, idx);
    [fo(idx, :), mLmS(idx), fStruct(idx)] = optimMLMS(e3, ...
        tmp(:, 1), shift1D(sp(tmp(:, 2), -1.5), 0, true), win, ...
        'preModel', 'Power Law', ...
        'postModel', 'Linear', ...
        'SScalculation', 'Decoupled', ...
        'sigModel', 'GLmix', ...
        'xlim', [695 745], ...
        'deblur', true, ...
        'shift', true, ...
        'algorithm', 'active-set');
    close(gcf);
end
```

```
Iteration 12
Iteration 11
Iteration 10
Iteration 09
Iteration 08
Iteration 07
Iteration 06
Iteration 05
Iteration 04
Iteration 03
Iteration 02
Iteration 01
```

```
toc;
```

Elapsed time is 21.051279 seconds.

```
% Plot the Orient Grain q-E map
LineEELS.grain = fStruct;
figure('Color', 'w');
imagesc(e3, abs(qy2(1:12)), [LineEELS.grain.EMCD]');
pbaspect([5 1 1]);
set(gca, 'YDir', 'normal');
set(gca, 'colormap', flipud(brewermap(1000, 'PuOr')));
set(gca, 'clim', [-0.04 0.04], 'xlim', [695 745]);
ylabel('q_y [mrad]');
xlabel('Energy [eV]');
title('q_y map for grain mask')
```

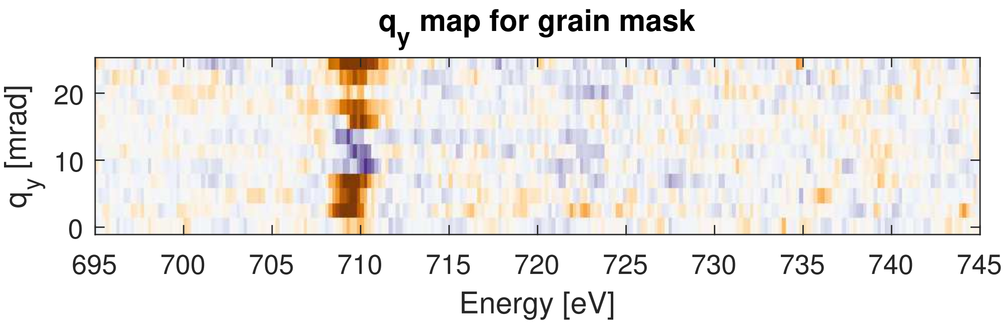

These final three maps are composited together to make figure 9 in the manscript.
